# Development of Carborane-Based Benzothiazole Analogues as Cannabinoid Receptor Type 2 (CB_2_R) Ligands

**DOI:** 10.1021/acsomega.5c06508

**Published:** 2025-08-13

**Authors:** Lea Ueberham, Aleksandr Kazimir, Winnie Deuther-Conrad, Evamarie Hey-Hawkins

**Affiliations:** † Centre for Biotechnology and Biomedicine (BBZ), Faculty of Chemistry, Institute of Bioanalytical Chemistry, 9180 Universität Leipzig, Deutscher Platz 5, 04103 Leipzig, Germany; ‡ Institute for Drug Discovery, Faculty of Medicine, Universität Leipzig, Brüderstraße 34, 04103 Leipzig, Germany; § Department of Experimental Neurooncological Radiopharmacy, Institute of Radiopharmaceutical Cancer Research, Helmholtz-Zentrum Dresden-Rossendorf (HZDR), Research Site Leipzig Permoserstraße 15, 04318 Leipzig, Germany; ∥ Department of Chemistry, Babeş-Bolyai University, Str. Arany Janos Nr. 11, RO-400028 Cluj-Napoca, Romania

## Abstract

The cannabinoid receptor type 2 (CB_2_R) is upregulated in the brain under pathological conditions. To distinguish between the healthy and disease states, Positron Emission Tomography (PET), as a noninvasive imaging technique, is employed, for which suitable highly affine and selective CB_2_R radioligands are required. The benzothiazole scaffold is a promising core structure that has been modified with different substituents. Recently, we have reported naphthyridinone- and thiazole-based carborane-substituted CB_2_R ligands and investigated the first carborane-based CB_2_R radiotracer **[** ^**18**^ **F]­LUZ5-** ***d***_**8**_ in preliminary biological tests. Carboranes are cluster compounds that are used as hydrophobic surrogates in drug design. We here report the synthesis, characterization, binding affinity data and docking results of three promising isomeric carborane-substituted benzothiazole-based CB_2_R ligands. The *ortho*-, *meta*- and *para*-carborane derivatives exhibit a nanomolar affinity and high selectivity toward CB_2_R, with the *meta*-carborane derivative being the most affine compound experimentally and in docking studies.

## Graphical abstract

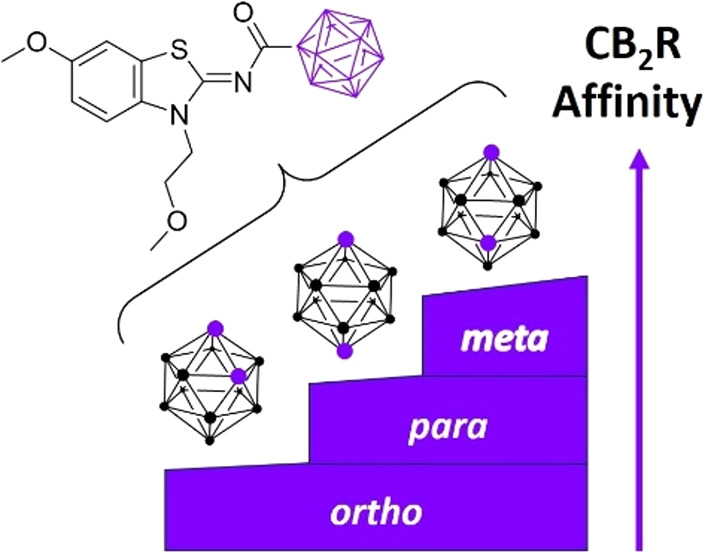

## Introduction

The cannabinoid receptors type 1 (CB_1_R) and type 2 (CB_2_R) are part of the endocannabinoid system (ECS), together with endogenous ligands, like anandamide and 2-arachidonoylgylcerol, and enzymes for their synthesis and degradation.^1^ Both receptors are involved in physiological and pathological conditions^2–5^ and share a high degree of similarity regarding their transmembrane domains.^6,7^ While the CB_1_R is expressed to a high degree in the central nervous system and brain,^2,5,7,8^ the CB_2_R is mainly attached to the immune system^2,5,8,9^ and is upregulated under pathological conditions in the brain.^3,10–12^ Because of its involvement in neurodegenerative diseases, inflammation and cancer, the CB_2_R is a target of interest.^5,7,10,13^ Positron Emission Tomography (PET), a noninvasive imaging method,^14,15^ also used in the investigation of cancer,^15,16^ is a fitting diagnostic tool to distinguish between healthy and disease states.^14,15,17^ A suitable CB_2_R radioligand must have a high selectivity and affinity toward CB_2_R, a high metabolic stability and must be able to pass the blood-brain-barrier (BBB).^4,13,14,18–20^ To date, there is no suitable CB_2_R ligand or radioligand for PET imaging routinely used in clinics.^13,14,18,21–23^

There are reports of some interesting compounds, like 2-oxoquinoline-based **[** ^**11**^ **C]­NE40** (**L1**, Figure 1) from Evens et al.^24,25^ and (dimethyl)­thiazole-based **[** ^**11**^ **C]­MDTC** (**L9**, Figure 2)^26^ from Horti et al.^27^ Both compounds have even been tested in humans.^26,28,29^ Other radioligands for the CB_2_R that have been published in the last years are indole-based **[** ^**18**^ **F]­DM102** ^30^ (**L3**, Figure 1) and **[** ^**18**^ **F]­RM365** ^31^ (**L4**, Figure 1), or the trisubstituted pyridine-based **[** ^**18**^ **F]­RoSMA-18-** ***d***_**6**_ ^32^ (**L2**, Figure 1). The latter was recently confirmed for a phase I clinical trial (NCT05880563, ClinicalTrials.gov). Furthermore, naphthyridinones with different substituents have been reported by Ferrarini et al.,^33^ Manera et al.,^34–36^ Lucchesi et al.,^12^ Pascali et al.,^37^ Gündel et al. (**[** ^**18**^ **F]­LU13**, **L5**, Figure 1)^38^, Teodoro et al. (**[** ^**18**^ **F]­LU14**, **L6**, Figure 1),^39^ as well as by us (carborane-substituted **L7**, Figure 1).^40^

**Figure 1:**
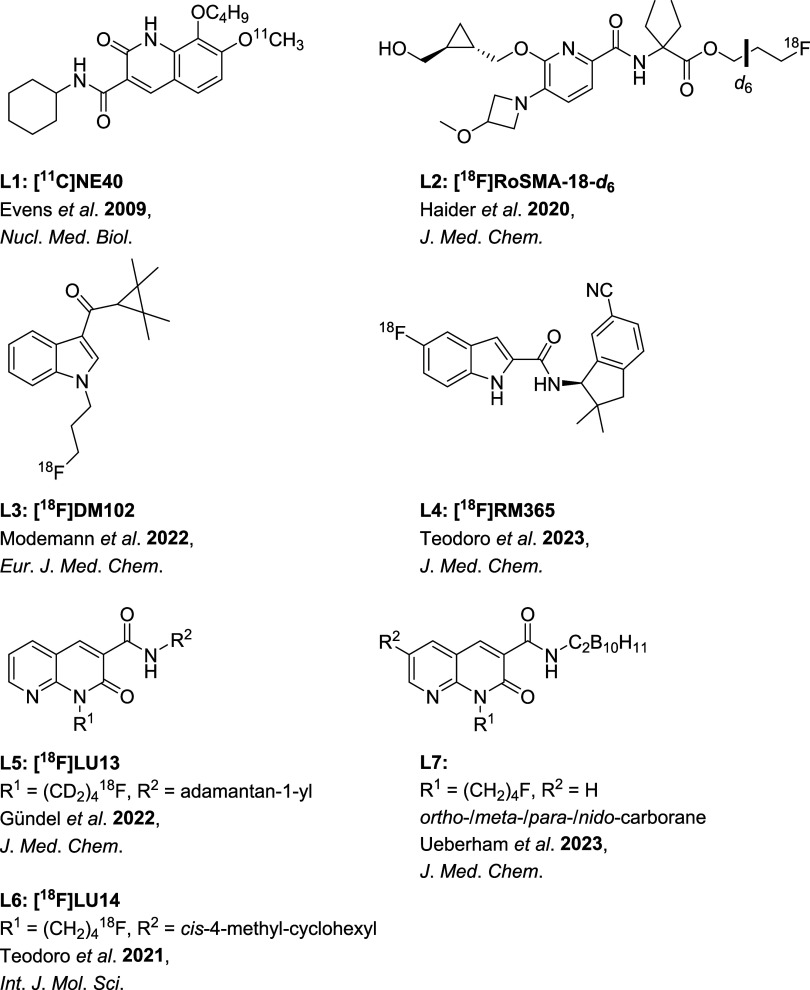
Selection of CB_2_R (radio)­ligands.^24,30–32,38–40^

**Figure 2:**
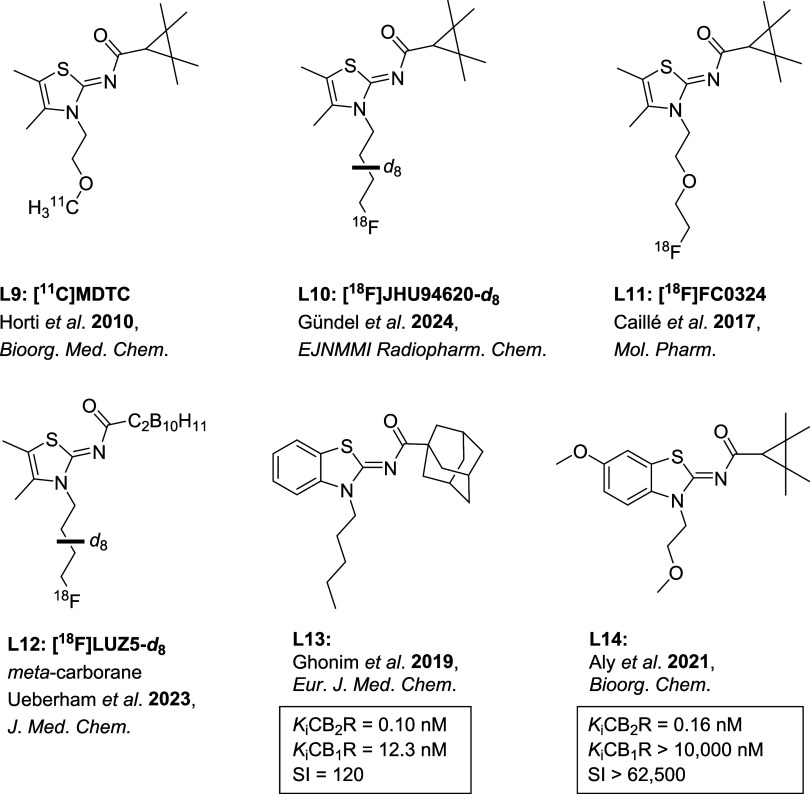
Selection of (benzo)­thiazole-based CB_2_R (radio)­ligands.^20,27,40,53–55^

Carboranes (*closo*-dicarbadodecaboranes­(12)) are icosahedral hydrophobic cluster compounds with ten BH and two CH units (Scheme S1, Supporting Information) that can be divided into *ortho-*, *meta-* and *para-*carborane, depending on the relative position of the two CH moieties.^41,42^ Carboranes can be substituted in three dimensions, making them superior to benzene^43^ and allowing to obtain finely tuned compounds, especially in the medicinal context.^43^ Another benefit is the (expected) high metabolic stability of carboranes *in vivo*.^40,43–47^ Furthermore, carboranes can be involved in noncovalent interactions, which can be favorable in biological surroundings.^43^ Carboranes have been used in the context of multiple medicinal applications, as substituents in COX-2 inhibitors or adenosine derivatives for instance.^48–51^ The carborane-substituted dimethylthiazole-based radiotracer **[** ^**18**^ **F]­LUZ5-** ***d***_**8**_ (**L12**, Figure 2),^40^ reported by us, showed an affinity in the subnanomolar range and an improved metabolic stability *in vivo* in rats and mice compared to the purely organic analogues **[** ^**18**^ **F]­JHU94620** ^52^ and **[** ^**18**^ **F]­FC0324** (**L11**, Figure 2).^53^ The *meta*-carborane derivative^40^ was in rat brain 30 min *p.i*., even more metabolically stable then the recently reported **[** ^**18**^ **F]­JHU94620-** ***d***_**8**_ (**L10**, Figure 2).^20^ Ghonim et al. have published interesting investigations regarding the structure–activity relationships of thiazole- and benzothiazole-based CB_2_R ligands.^54^ The so designed compound **L13** (Figure 2) showed a remarkable affinity toward CB_2_R, but an insufficient selectivity.^54^ Following up on this work, Aly et al.^55^ prepared different highly substituted benzothiazole analogues as CB_2_R ligands and could improve the selectivity while retaining a very high affinity (**L14**, Figure 2).

We here report an extension of our previous work on carborane-based CB_2_R ligands^40^ to another promising CB_2_R ligand, namely the benzothiazole scaffold, because of its similarity to the thiazole core and based on the promising high affinity and selectivity that could be reached for benzothiazole-based compounds by Aly et al.^55^ The synthesis, characterization, *in vitro* evaluation of binding affinity toward CB_1_R and CB_2_R and *in silico* studies of binding affinity toward CB_2_R of *ortho*-, *meta*- and *para*-carborane-substituted benzothiazole derivatives are described.

## Results and Discussion

### Synthesis

The reaction of compound **1** ^55^ with the respective carboranyl acid chloride **A**_***o***, ***m***, ***p***_ ^56–58^ adapted from Richter et al.^59^ yielded the target compounds **2**_***o***, ***m***, ***p***_ (Scheme 1; for more information see the Supporting Information). Compounds **2**_***o***_, **2**_***m***_ and **2**_***p***_ were purified by column chromatography and characterized by 1D and 2D NMR spectroscopy and ESI-HRMS. The purity and stability were evaluated by HPLC-MS (see Supporting Information).

**Scheme 1:**
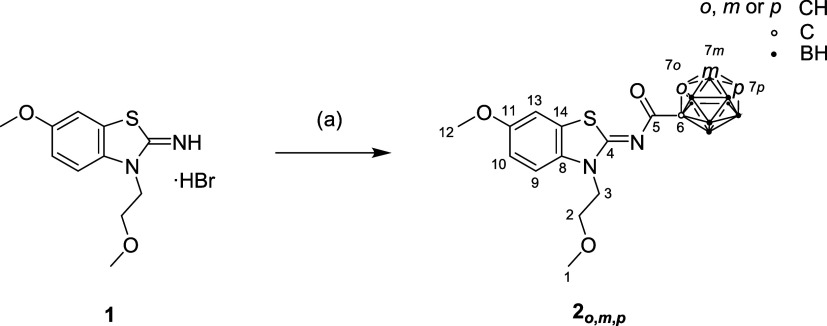
Synthesis of Target Compounds **2** *_**o**_*, **2** *_**m**_*, **2** *_**p**_* ^a^

a Reagents and conditions: (a) (i) dry CH_3_CN, reflux; (ii) respective 1-C­(O)­Cl-C_2_B_10_H_11_ (**A**_*o*,*m*,*p*_), 1 h 45 min – 2 h 30 min; (iii) (dry) NEt_3_, reflux, 20 h 30 min – 24 h (in a closed vessel).

### Biological Studies

The binding affinity of **2**_***o***_, **2**_***m***_ and **2**_***p***_ toward CB_1_R and CB_2_R have been determined in *in vitro* competitive assays with crude membrane preparations from Chinese hamster ovary (CHO) cells stably transfected with human CB_1_R or CB_2_R. The assays have been performed using **[** ^**3**^ **H]­SR141716A** (for CB_1_R, Figure S26, Supporting Information) and **[** ^**3**^ **H]­WIN55212–2** (for CB_2_R, Figure S26, Supporting Information). The results are shown in Table 1.

**Table 1:** Binding Affinities of Compounds **2** *_**o**_*, **2** *_**m**_* and **2** *_**p**_*

compound	*K*_i_CB_2_R (nM) mean^a^	*K*_i_CB_1_R (nM) mean^a^	selectivity index (SI)^b^
**2**_***o***_	7.37	76815	10426
**2**_***m***_	2.47	42270	17102
**2**_***p***_	3.17	8666	2734

a Mean of two values.

b SI = *K*_i_CB_1_R/*K*_i_CB_2_R.

Compounds **2**_***o***_, **2**_***m***_ and **2**_***p***_ show a high affinity toward CB_2_R in the nanomolar range and a high selectivity index (SI). The *meta*-carborane derivative **2**_***m***_, is the most affine compound, with a mean *K*_i_CB_2_R of 2.47 nM. The benzothiazole derivatives are less potent than the carborane-based CB_2_R ligand **[** ^**18**^ **F]­LUZ5-** ***d***_**8**_,^40^ but show the same trend as had been observed for the corresponding thiazole carborane derivatives, with the *meta*-carborane derivative being the most affine compound, followed by the *para*- and *ortho*-carborane derivatives.^40^ While some organic compounds showed a higher affinity (e.g., Aly et al., **L14**, Figure 2, *K*_i_CB_2_R = 0.16 nM),^55^ our results indicate that by introducing a carborane unit in benzothiazole derivatives a high affinity for CB_2_R is retained with potentially improved metabolic stability.

### Docking Results

The binding affinities of **2**_***o***_, **2**_***m***_ and **2**_***p***_ toward CB_2_R were determined based on *in silico*-constructed structures (Table 2). Furthermore, it was evaluated, whether specific ligand–amino acid interactions play a crucial role in receptor activation. If the binding of a compound stabilizes the receptor in an inactive conformational state, this compound may act as an antagonist. For human CB_2_R, especially significant are the interaction with Trp258,^60^ a toggle switch, or conformational changes in the binding pocket that may influence Trp258.

**Table 2:** Binding Energy Values Determined by Docking of Compounds **2** *_**o**_*, **2** *_**m**_*, **2** *_**p**_* Into Human CB_2_R (PDB structure: 5zty^61^)

compound	binding energy values in kcal/mol
**2_*o*_**	–9.46
**2_*m*_**	–10.25
**2_*p*_**	–9.22

The top-ranked binding poses of the three isomers **2**_***o***_, **2**_***m***_, **2**_***p***_ revealed interactions with amino acid residues Val95, Ile92, Phe165, Phe88, Ser72, and Val391 and a similar alignment of these structures within the binding pocket. Interestingly, the carborane cluster of **2**_**m**_ was positioned deeper within the hydrophobic pocket, while the benzothiazole moiety was stabilized by interactions with Lys408, Lys91, and Phe76.

Binding energy calculations indicated that the *meta*-carborane derivative exhibited the highest affinity, followed by the *ortho*- and the *para*-carborane derivatives (*meta* > *ortho* > *para*). However, none of the structures showed direct interactions with the Trp258 toggle switch^60^ (Figure 3).

**Figure 3:**
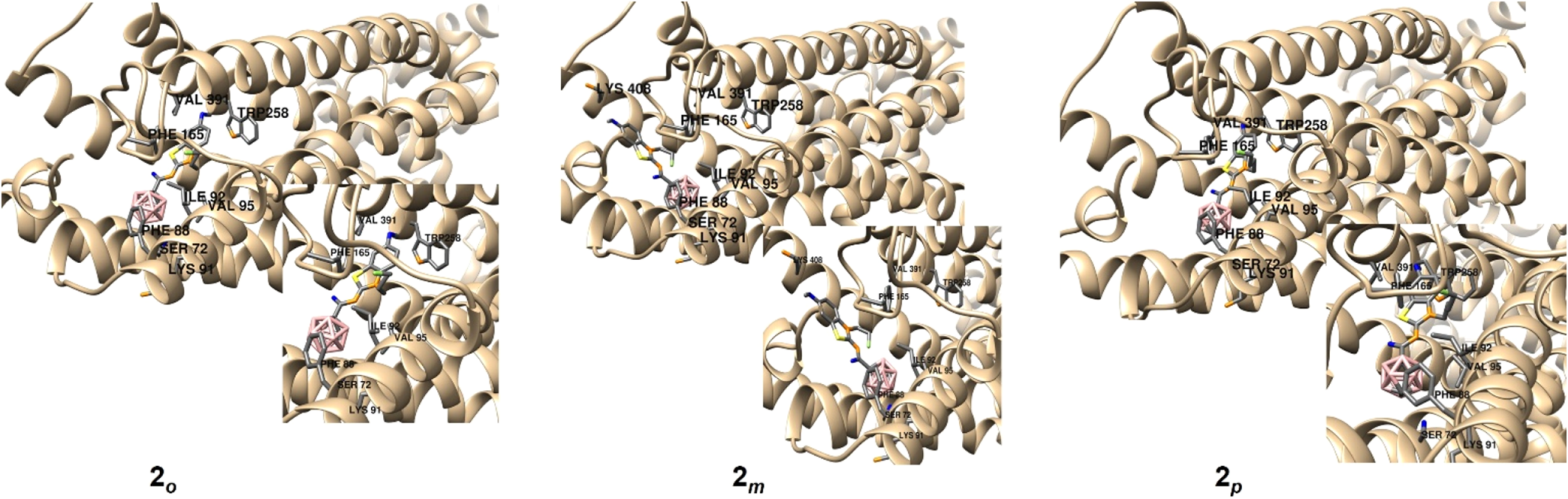
*In silico* investigation of the binding modes of compounds **2** *_**o**_*, **2** *_**m**_* and **2** *_**p**_* in human CB_2_R based on docking. The highest ranked docked positions are shown together with amino acid residues.

The docking results partially align with the experimentally obtained binding affinity data. In both cases, the *meta*-carborane derivative **2**_***m***_ was the most affine compound. The deeper orientation of the carborane cluster in the best-ranked pose of **2**_***m***_ within the hydrophobic pocket, along with noncovalent interactions between benzothiazole and specific amino acid residues (π-π, C–H···π-interactions, H-bonds) (see Figure 3) likely contribute to the binding affinity. In contrast to the calculated values, the experimentally obtained data show that the *para*-carborane derivative **2**_***p***_ has an improved affinity toward CB_2_R compared to the *ortho*-carborane derivative **2**_***o***_. This discrepancy can be explained by the limitations of the scored binding energies, as enthalpic and entropic terms require higher level of theory. Additional information is available in the Supporting Information.

## Conclusions

We have shown that carboranes are suitable hydrophobic substituents in CB_2_R ligands. Three carborane-based benzothiazole derivatives have been synthesized, fully characterized and the binding affinities toward CB_1_R and CB_2_R have been determined *in vitro*. The high affinities toward CB_2_R in the nanomolar range and high selectivities make them ideal candidates for further (metabolic) investigations. The *meta*-carborane derivative **2**_***m***_ is the most promising ligand in the investigated set of compounds, and *in silico* data corroborate this finding. Our results further confirm that there is an influence of the relative position of the two C atoms (one of which is a CH moiety) in carboranes on the affinity and selectivity toward CB_2_R.

## Experimental Section

### General Information

All carborane-based reactions were carried out under nitrogen atmosphere, using Schlenk technique. Acetonitrile was dried over calcium hydride, distilled and stored over molecular sieves (4 or 5 Å). Triethylamine was purified and dried as described in Purification of Laboratory Chemicals.^62^ Compounds **A**_***o***, ***m***, ***p***_ were synthesized according to the literature.^56–58^ Compound **1** was prepared as described by Aly et al.^55^ All other solvents and chemicals were commercially available and used without further purification. Reaction progress and purification were monitored by thin-layer chromatography (TLC) using precoated silica gel 60 F_254_ Alugram plates (Xtra SIL G) from Macherey-Nagel (Düren, Germany). Carborane-containing TLC spots were stained with a solution of 5–10% PdCl_2_ in methanol. Chromatography was performed in air, with silica gel (60 Å, 0.035–0.070 mm particle diameter) in an automated fashion with Isolera-4 and ELSD 1080 (Biotage) with commercially available solvents. NMR spectra were recorded with a Varian MERCURYplus 400 spectrometer or with Bruker AVANCE III HD 400 or Avance DRX 400 spectrometer from Bruker (Billerica, MA). Measurements were performed at 400 MHz (^1^H), 128 MHz (^11^B) and 101 MHz (^13^C). Chemical shifts (δ) are given in parts per million (ppm). ^1^H and ^13^C NMR spectra were referenced to internal deuterated solvent and ^11^B­{^1^H} NMR spectra to the Ξ scale.^63^ The deuterated solvent CDCl_3_ was purchased from Eurisotop (Saint-Aubin, France) with a deuteration rate of 99.80%. High-resolution mass spectrometry (HRMS) was conducted in positive ion mode with an ESI-TOF microTOF instrument from Bruker Daltonik GmbH (Bremen, Germany) with CH_3_CN solutions of the compounds. The simulation of mass spectra was carried out with the Web-based MS online tool of Scientific Instrument Service (SISweb, Palmer, MA).^64^ The analysis of NMR and MS data was done with MestReNova version 14.1.0.^65^

### Chemical Synthesis

#### General Procedure for the Synthesis of 2_*o*,*m*,*p*_

The respective acid chloride **A**_***o***, ***m***, ***p***_ was added to a solution of **1** in dry CH_3_CN (3–8 mL) under reflux. (Dry) triethylamine was added after 1 h 45 min to 2 h 40 min (in a closed vessel) and the solution was stirred for 20 h 30 min to 24 h (in a closed vessel) under reflux.

#### (*Z*)-*N*-(6-Methoxy-3-(2-methoxyethyl)­benzo­[*d*]­thiazol-2­(3*H*)-ylidene)­(1,2-*closo*-dicarba-dodecaborane)­carboxamide (2_*o*_)

Reagents and conditions: **1** (semicrude): 436 mg, 1.37 mmol, 1.00 eq.; **A**_***o***_ in toluene (added in two portions): 2.5 mL, 346.2 mg, 1.68 mmol, 0.67 M, 1.23 eq.; dry NEt_3_: 0.5 mL, 365 mg, 3.61 mmol, 2.64 eq. The solvent was removed directly after the reaction. The crude product was purified by column chromatography: twice *n*-hexane/EtOAc, 87:13 (v/v) → 100% EtOAc, then *n*-hexane/EtOAc, 90:10 (v/v) → 77% EtOAc and *n*-hexane/EtOAc, 95:5 (v/v) → 41% EtOAc and *n*-hexane/CH_2_Cl_2_, 3:1 (v/v) → 100% CH_2_Cl_2_. In the process of purification via column chromatography, fractions from a previous reaction have been purified together with fractions of this reaction, to gain enough pure compound for full characterization and *in vitro* tests. Therefore, no yield can be determined. Compound **2**_***o***_ was obtained as a white solid. It was only enough compound purified to fully characterize the compound and determine the *in vitro* binding affinities.

^1^H NMR (400 MHz, CDCl_3_) δ 0.51–3.42 (br, 10H, B**H**), 3.31 (s, 3H, **1-**OC**H**_**3**_), 3.79 (t, ^3^ *J* = 5.2 Hz, 2H, **2-**C**H**_**2**_), 3.87 (s, 3H, **12-**OC**H**_**3**_), 4.32 (s, 1H, **7** ***o*** **-**C**H**_**Cluster**_), 4.53 (t, ^3^ *J* = 5.3 Hz, 2H, **3-**C**H**_**2**_), 7.08 (dd, ^3^ *J* = 9.0, ^4^ *J* = 2.5 Hz, 1H, **10-**C**H**_**ar**_), 7.18 (d, ^4^ *J* = 2.5 Hz, 1H, **13-**C**H**_**ar**_), 7.47 (d, ^3^ *J* = 9.0 Hz, 1H, **9-**C**H**_**ar**_); ^13^C­{^1^H} NMR (101 MHz, CDCl_3_) δ 46.9 (**3-C**H_2_), 56.1 (**12-**O**C**H_3_), 56.8 (**7** ***o*** **-C**H_Cluster_), 59.3 (**1-**O**C**H_3_), 69.9 (**2-C**H_2_), 106.2 (**13-C**H_ar_), 114.4 (**9-C**H_ar_), 116.0 (**10-C**H_ar_), 127.5 (**14-C**_**quart**_), 131.2 (**8-C**_**quart**_), 157.6 (**11-C**_**quart**_), 167.7 (**4-C**_**quart**_), 167.9 (**5-C**O); ^11^B­{^1^H} NMR (128 MHz, CDCl_3_) δ −13.7 (s, 2B), −11.7 (s, 6B), −9.3 (s, 1B), −3.5 (s, 1B); HRMS (ESI+) *m*/*z* for C_14_H_25_B_10_N_2_O_3_S [M + H]^+^ 409.2596, calcd 409.2589.

#### (*Z*)-*N*-(6-Methoxy-3-(2-methoxyethyl)­benzo­[*d*]­thiazol-2­(3*H*)-ylidene)­(1,7-*closo*-dicarba-dodecaborane)­carboxamide (2_*m*_)

Reagents and conditions: **1** (semicrude): 249 mg, 0.780 mmol, 1.00 eq.; **A**_***m***_: 185 mg, 0.895 mmol, 1.15 eq.; NEt_3_: 0.18 mL, 131.4 mg, 1.30 mmol, 1.66 eq. The volume of the solution containing the product was reduced under reduced pressure and CH_2_Cl_2_ was added to the warm residue. The resulting white precipitate was filtered off and the solution was purified by column chromatography: *n*-hexane/EtOAc, 95:5 (v/v) → 70% EtOAc. The solid was dissolved in isopropanol/EtOH and the obtained crystals were isolated by filtration. Compound **2**_***m***_ was obtained as a white solid. It was only enough compound purified to fully characterize the compound and determine the *in vitro* binding affinities.

^1^H NMR (400 MHz, CDCl_3_) δ 0.55 −4.10 (br, 10H, B**H**), 2.99 (s, 1H, **7** ***m*** **-**C**H**_**Cluster**_), 3.31 (s, 3H, **1-**OC**H**_**3**_), 3.79 (t, ^3^ *J* = 5.3 Hz, 2H, **2-**C**H**_**2**_), 3.86 (s, 3H, **12-**OC**H**_**3**_), 4.51 (t, ^3^ *J* = 5.4 Hz, 2H, **3-**C**H**_**2**_), 7.05 (dd, ^3^ *J* = 9.0, ^4^ *J* = 2.6 Hz, 1H, **10-**C**H**_**ar**_), 7.16 (d, ^4^ *J* = 2.5 Hz, 1H, **13-**C**H**_**ar**_), 7.43 (d, ^3^ *J* = 9.0 Hz, 1H, **9-**C**H**_**ar**_); ^13^C­{^1^H} NMR (101 MHz, CDCl_3_) δ 46.7 (**3-C**H_2_), 54.5 (**7** ***m*** **-C**H_Cluster_), 56.1 (**12-**O**C**H_3_), 59.3 (**1-**O**C**H_3_), 69.9 (**2-C**H_2_), 106.3 (**13-C**H_ar_), 114.0 (**9-C**H_ar_), 115.7 (**10-C**H_ar_), 127.7 (**14-C**_**quart**_), 131.2 (**8-C**_**quart**_), 157.3 (**11-C**_**quart**_), 167.5 (**4-C**_**quart**_), 170.8 (**5-C**O); ^11^B­{^1^H} NMR (128 MHz, CDCl_3_) δ −15.5 (s, 2B), −13.6 (s, 2B), −11.0 (s, 4B), −7.9 (s, 1B), −4.7 (s, 1B); HRMS (ESI+) *m*/*z* for C_14_H_25_B_10_N_2_O_3_S [M + H]^+^ 409.2589, calcd 409.2589.

#### (*Z*)-*N*-(6-Methoxy-3-(2-methoxyethyl)­benzo­[*d*]­thiazol-2­(3*H*)-ylidene)­(1,12-*closo*-dicarba-dodecaborane)­carboxamide (2_*p*_)

Reagents and conditions: **1** (semicrude): 375 mg, 1.12 mmol, 1.00 eq.; **A**_***p***_: 273 mg, 1.32 mmol, 1.18 eq.; NEt_3_: 0.36 mL, 263 mg, 2.60 mmol, 2.32 eq. The triethylamine was added in two portions, one after 2 h 40 min and the second after another 18 h 30 min. Acentontrile was added, the suspension was filtered, the solvent of the solution containing the product was removed under reduced pressure and EtOAc was added. The resulting white precipitate was filtered off and the solution was purified by column chromatography: *n*-hexane/EtOAc, 87:13 (v/v) → 60% EtOAc. Compound **2**_***p***_ was obtained as an off-white solid. It was only enough compound purified to fully characterize the compound and determine the *in vitro* binding affinities.

^1^H NMR (400 MHz, CDCl_3_) δ 0.53–3.43 (br, 10H, B**H**), 2.77 (s, 1H, **7** ***p*** **-**C**H**_**Cluster**_), 3.31 (s, 3H, **1-**OC**H**_**3**_), 3.76 (t, ^3^ *J* = 5.3 Hz, 2H, **2-**C**H**_**2**_), 3.84 (s, 3H, **12-**OC**H**_**3**_), 4.46 (t, ^3^ *J* = 5.4 Hz, 2H, **3-**C**H**_**2**_), 7.02 (dd, ^3^ *J* = 9.0, ^4^ *J* = 2.5 Hz, 1H, **10-**C**H**_**ar**_), 7.12 (d, ^4^ *J* = 2.5 Hz, 1H, **13-**C**H**_**ar**_), 7.40 (d, ^3^ *J* = 9.0 Hz, 1H, **9-**C**H**_**ar**_); ^13^C­{^1^H} NMR (101 MHz, CDCl_3_) δ 46.6 (**3-C**H_2_), 56.1 (**12-**O**C**H_3_), 59.3 (**1-**O**C**H_3_), 61.1 (**7** ***p*** **-C**H_Cluster_), 69.9 (**2-C**H_2_), 106.3 (**13-C**H_ar_), 113.8 (**9-C**H_ar_), 115.5 (**10-C**H_ar_), 127.7 (**14-C**_**quart**_), 131.2 (**8-C**_**quart**_), 157.1 (**11-C**_**quart**_), 167.4 (**4-C**_**quart**_), 170.1 (**5-C**O); ^11^B­{^1^H} NMR (128 MHz, CDCl_3_) δ −13.1 (s, 5B), −15.5 (s, 5B); HRMS (ESI+) *m*/*z* for C_14_H_25_B_10_N_2_O_3_S [M + H]^+^ 409.2589, calcd 409.2589.

### Determination of Purity

The purity of the compounds **2**_***o***, ***m***, ***p***_ was determined with an HPLC-UV-MS system (UltiMate 3000 UHPLC System from Thermo Scientific, Germering, Germany, DAD detector: DAD-3000RS, coupled to MSQ Plus single quadrupole mass spectrometer from Thermo Scientific, Austin, TX), as published previously.^48^ The compounds were dissolved in CH_3_CN and measured with a Poroshell 120 EC-C_18_ column (100 mm × 3 mm, 2.7 μm) from Agilent Technologies (Waldbronn, Germany) at 25 °C with a flow of 0.7 mL/min. The gradient system consisted of LC-MS grade water +0.1% formic acid (eluent A) and CH_3_CN + 0.1% formic acid (eluent B). The gradient used was 40% eluent B (0–1.5 min), 40–100% (1.5–10 min), 100% eluent B (10–15 min), 40% eluent B (15–20 min). Prior to every measurement a blank run with pure CH_3_CN was performed. All compounds had a purity of >95% (Figures S19–S22, Supporting Information).

### Stability Measurements

The determination of stability was performed analogously to the purity determination and as previously published.^48^ Instead of CH_3_CN, a mixture of DMSO/H_2_O (1:1, v/v) was used as blank sample and as medium for the measurements of compounds **2**_***o***, ***m***, ***p***_. The measurements were performed directly after addition of water to the respective compound dissolved in DMSO and at varying time points up to 1 d (Figures S23–S25, Supporting Information).

### Binding Affinity

For the *in vitro* binding affinity determination toward CB_1_R and CB_2_R, membrane homogenates from Chinese hamster ovary cells (CHO) stably transfected with the human CB_1_R or CB_2_R have been used. The assays were performed according to an already published protocol.^66^

## Supplementary Material



## Data Availability

The data that support the findings of this study are available in the Supporting Information of this article.

## Notes

NMR spectra, HRMS-ESI spectra, purity and stability determination and docking data of compounds **2*_o_***, **2*_m_*** and **2*_p_*** (PDF)
