# Supplementary material for: Development of Carborane-Based Benzothiazole Analogues as Cannabinoid Receptor Type 2 (CB2R) Ligands
Source: ACS Omega. 2025 Aug 13;10(33):38264–71. doi: 10.1021/acsomega.5c06508 (PMC12392035; doi:10.1021/acsomega.5c06508)
Supplement: Supplementary file 1 [file ao5c06508_si_001.pdf]

Supporting Information

Development of Carborane-Based Benzothiazole Analogues as Cannabinoid Receptor Type 2 (CB_2R) Ligands

Lea Ueberham, [a] Aleksandr Kazimir, [b] Winnie Deuther-Conrad, [c] Evamarie Hey-Hawkins*[a, d]

[a] L. Ueberham, Prof. Dr. Dr. h.c. mult. E. Hey-Hawkins
Centre for Biotechnology and Biomedicine (BBZ)
Faculty of Chemistry
Institute of Bioanalytical Chemistry
Universität Leipzig
Deutscher Platz 5, 04103 Leipzig, Germany
E-mail: hey@uni-leipzig.de

[b] Dr. A. Kazimir
Institute for Drug Discovery
Faculty of Medicine
Universität Leipzig
Brüderstraße 34, 04103 Leipzig, Germany

[c] Dr. W. Deuther-Conrad
Department of Experimental Neurooncological Radiopharmacy
Institute of Radiopharmaceutical Cancer Research
Helmholtz-Zentrum Dresden-Rossendorf (HZDR),
Research Site Leipzig
Permoserstraße 15, 04318 Leipzig, Germany

[d] Prof. Dr. Dr. h.c. mult. E. Hey-Hawkins
Department of Chemistry
Babeș-Bolyai University
Str. Arany Janos Nr. 11
RO-400028 Cluj-Napoca, Romania

Contents

1 Experimental Section – Synthesis of Compounds 1 and $A_{o,m,p}$ S2

2 NMR Spectra of Compounds 2_o, 2_m and 2_p S2

3 HR-ESI Mass Spectra of Compounds 2_o, 2_m and 2_p S11

4 Determination of HPLC Purity of Compounds 2_o, 2_m and 2_p S13

5 Determination of the Stability of Compounds 2_o, 2_m and 2_p by HPLC S15

6 Docking Studies of Compounds 2_o, 2_m and 2_p S16

7 Chemical Structures of Compounds SR141716A and WIN55212-2 .... S17

References S17

1 Experimental Section – Synthesis of Compounds 1 and $A_{o,m,p}$

Compounds $\mathbf{A}_{o,m,p}$ have been synthesised in two steps, as reported by Choi *et al.*, Scholz *et al.* and Kasar *et al.*[1–3]. Compound **1** was prepared from commercially available starting materials as described by Aly *et al.*[4].

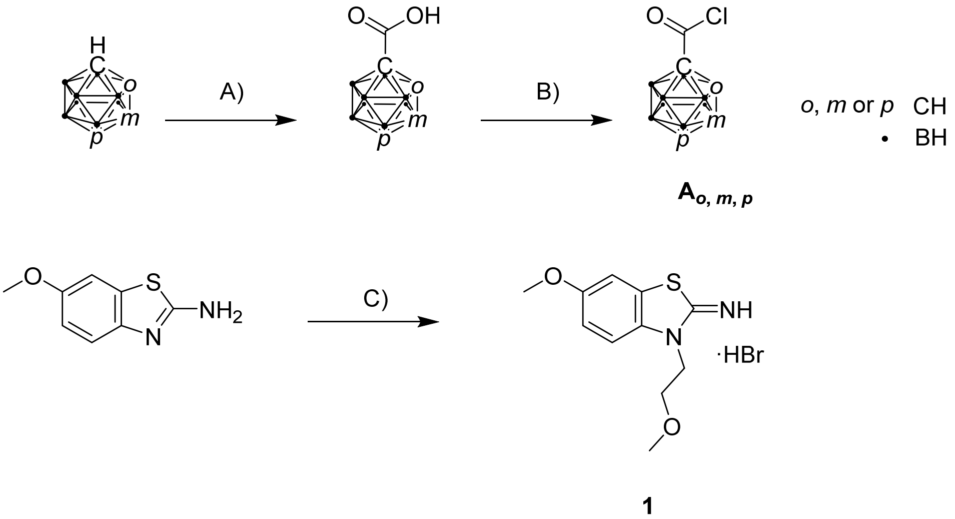

Scheme S1. Synthesis of compounds $\mathbf{A}_{o,m,p}$ and **1**. Reagents and conditions: (A) (i) *n*-butyllithium (*n*-BuLi), Et_2O, CO_2, rt, 16−21 h; (ii) HCl; (B) PCl_5, toluene, rt, 1 h; (C) (i) dry DMF, NaH, rt, 80 min, (ii) dry DMF, 2-bromoethyl methyl ether, 90 °C, 1 d.

2 NMR Spectra of Compounds 2_o, 2_m and 2_p

Compound $\mathbf{2}_o$

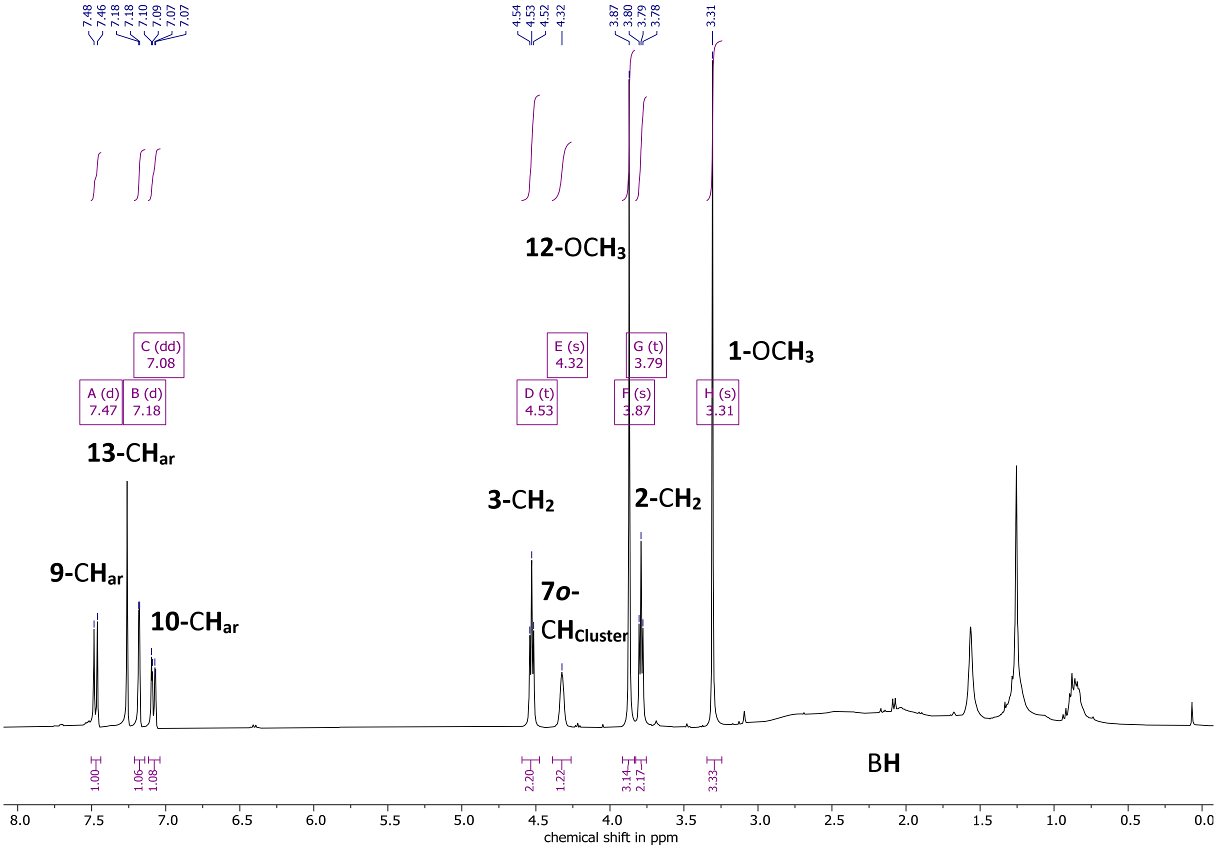

Figure S1. ^1H NMR spectrum of compound $\mathbf{2}_o$ in $CDCl_3$.

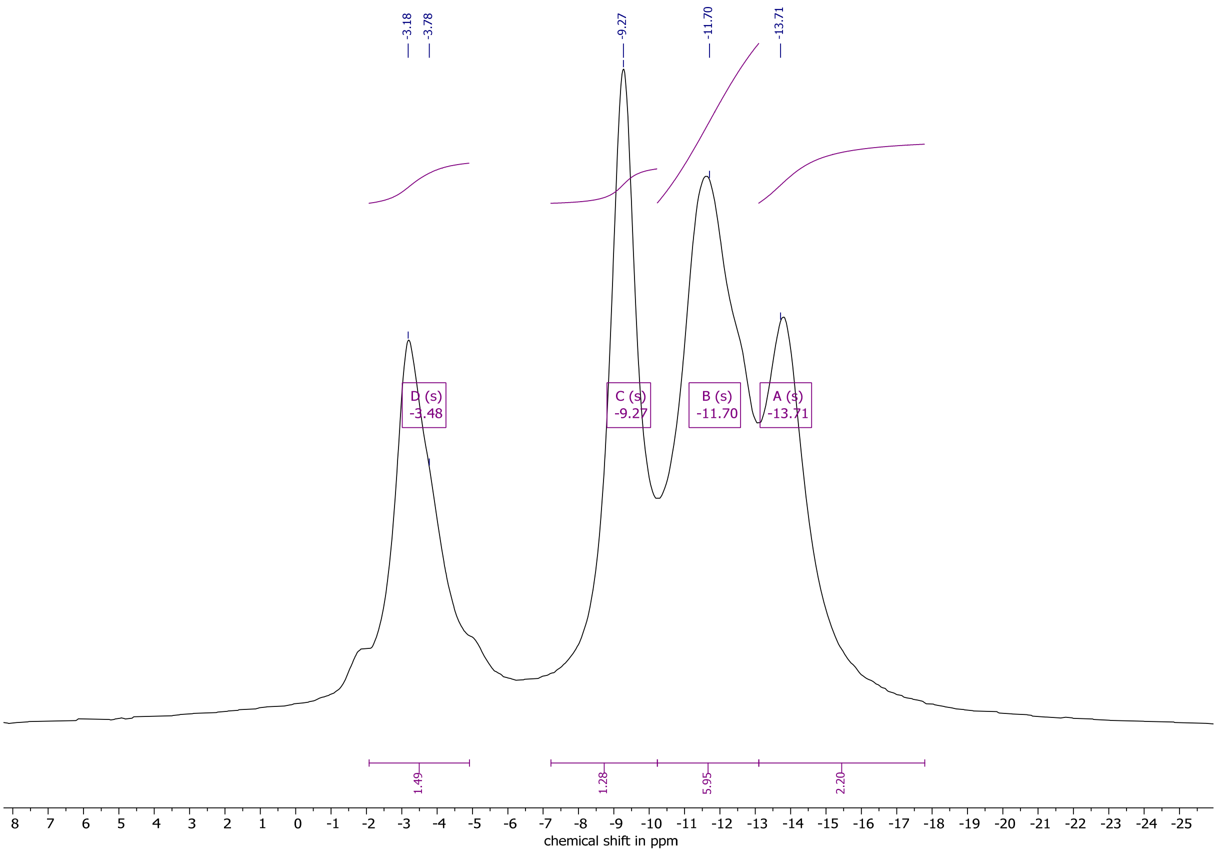

Figure S2. $^{11}B\{^{1}H\}$ NMR spectrum of compound **2*o*** in $CDCl_3$.

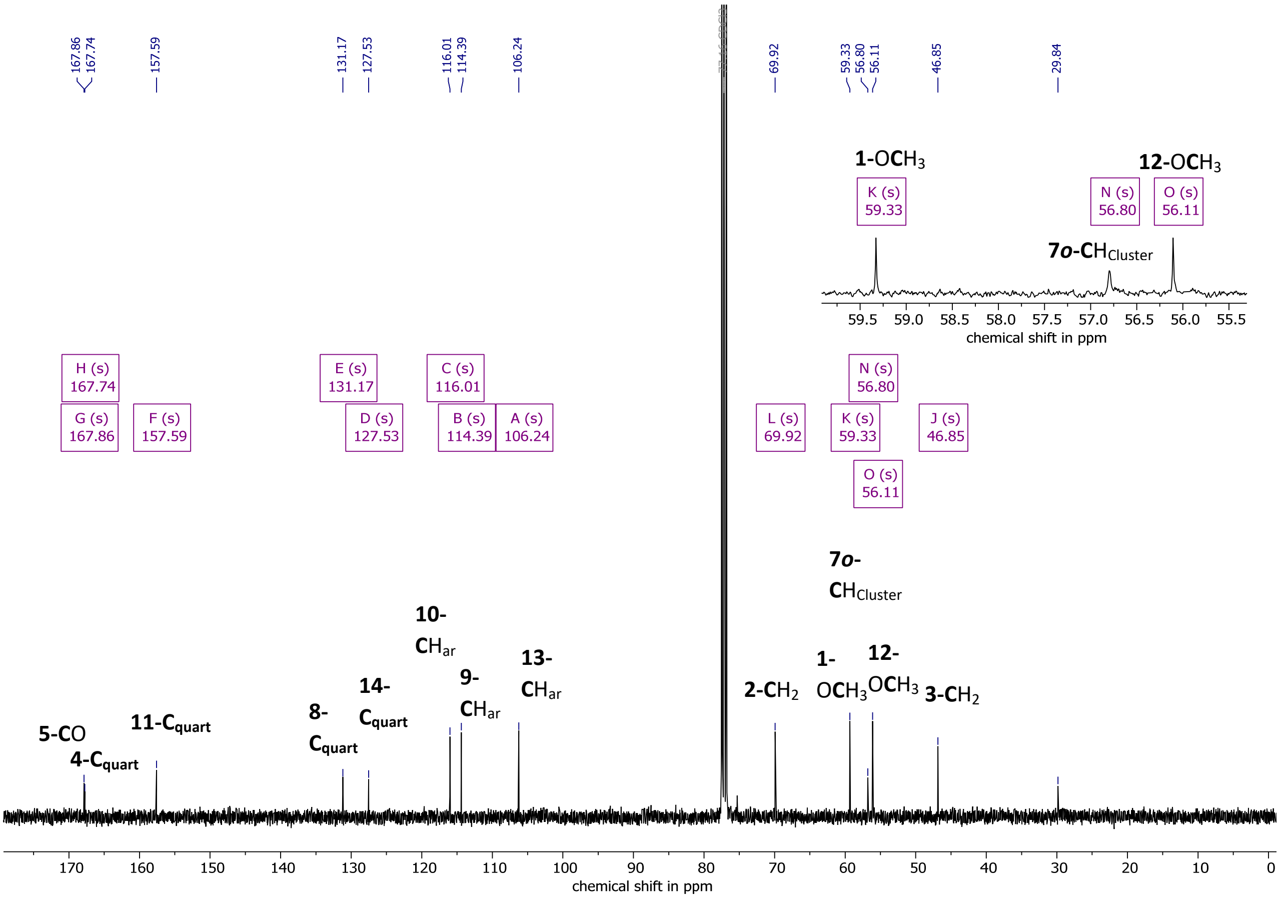

Figure S3. $^{13}C\{^{1}H\}$ NMR spectrum of compound **2*o*** in $CDCl_3$.

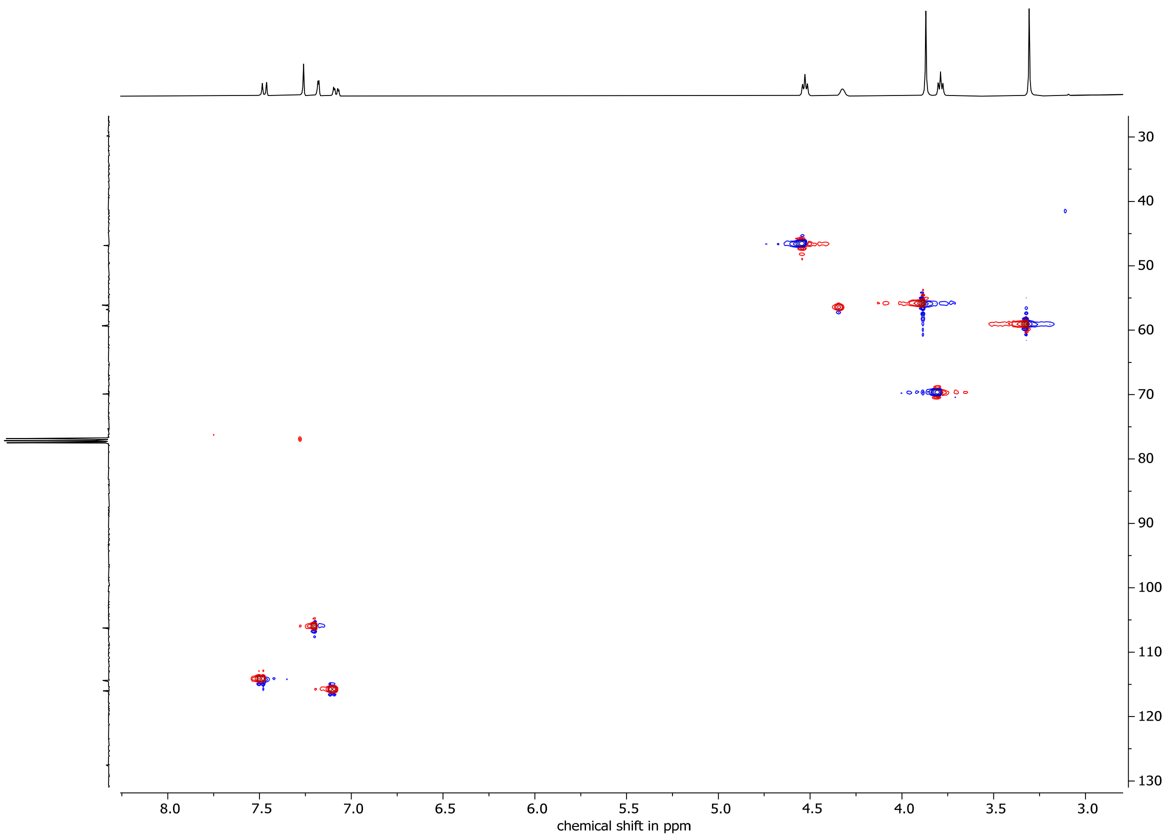

Figure S4. HSQC (^{1}H, ^{13}C) NMR spectrum of compound **2o** in CDCl3.

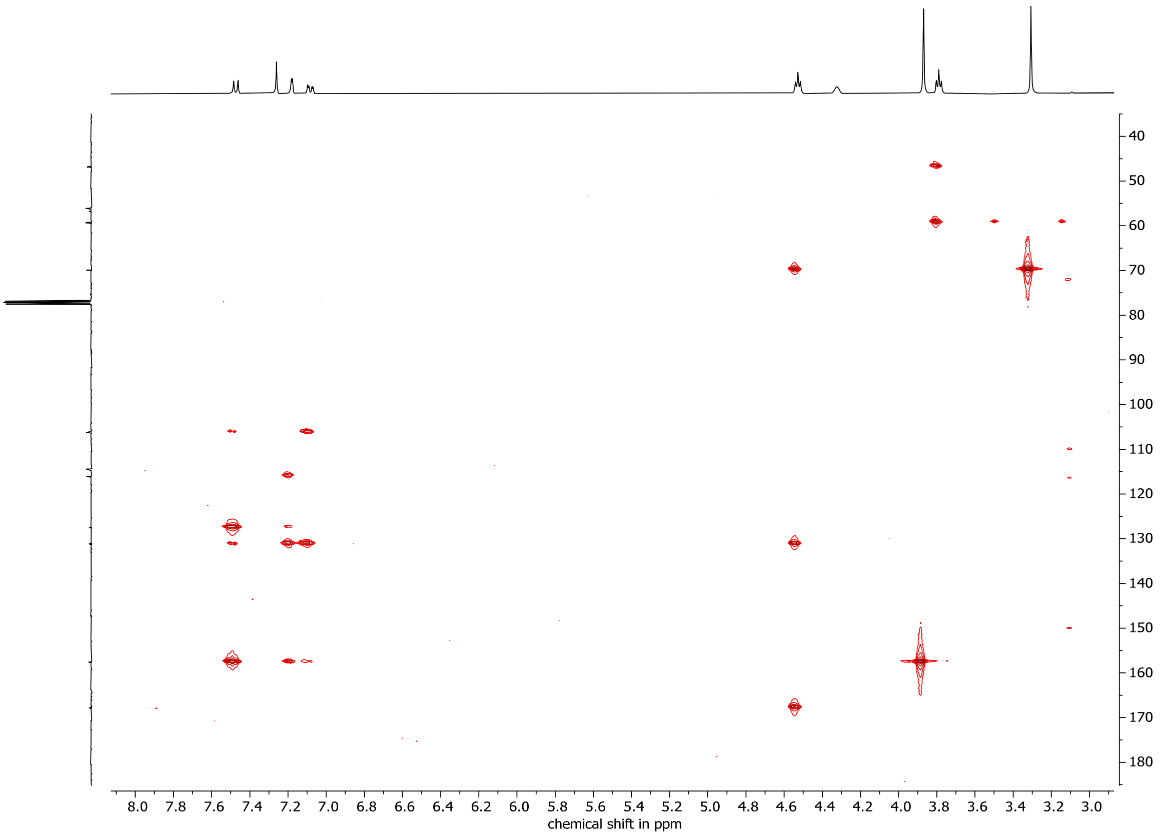

Figure S5. HMBC (^{1}H, ^{13}C) NMR spectrum of compound **2o** in CDCl3.

Compound **2*m***

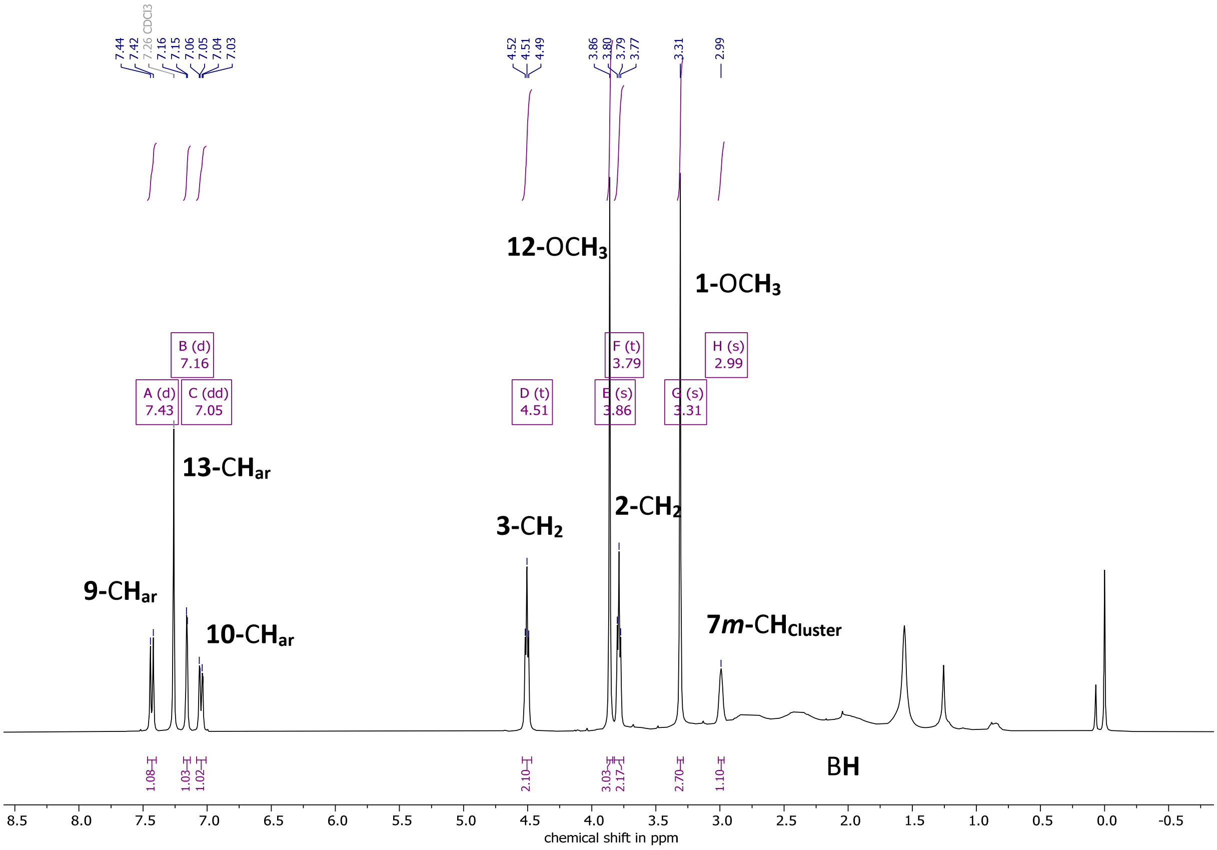

Figure S6. ^{1}H NMR spectrum of compound **2*m*** in $CDCl_3$.

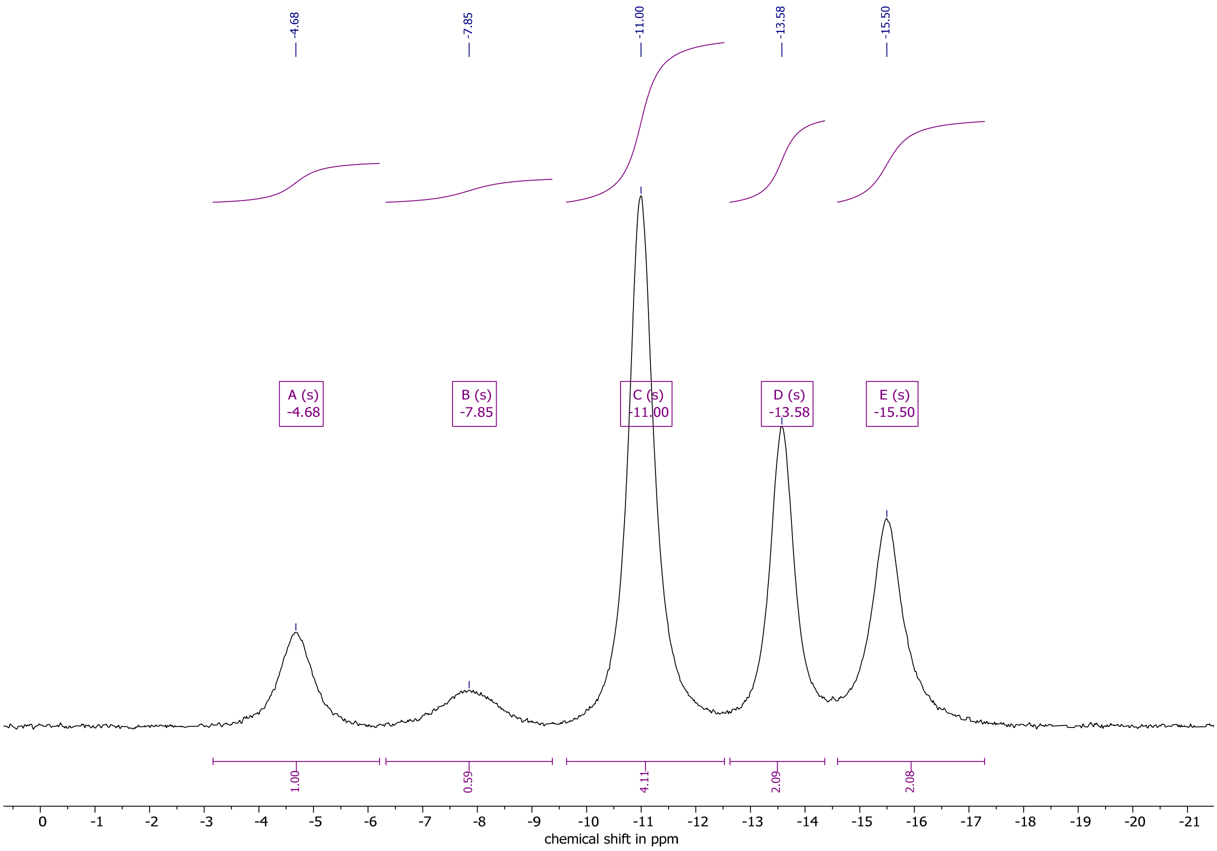

Figure S7. $^{11}B\{^{1}H\}$ NMR spectrum of compound **2*m*** in $CDCl_3$.

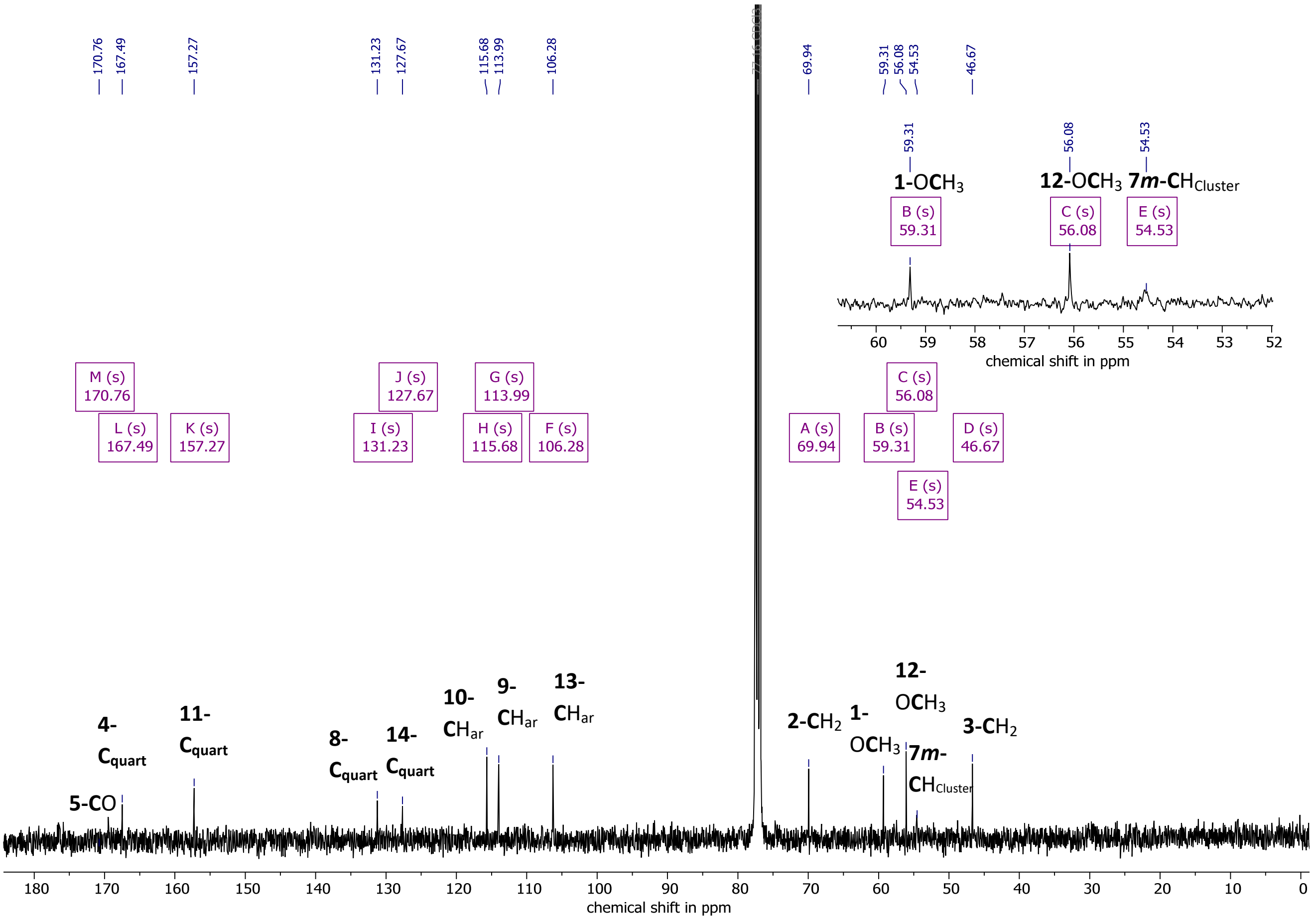

Figure S8. $^{13}C\{^1H\}$ NMR spectrum of compound **2*m*** in $CDCl_3$.

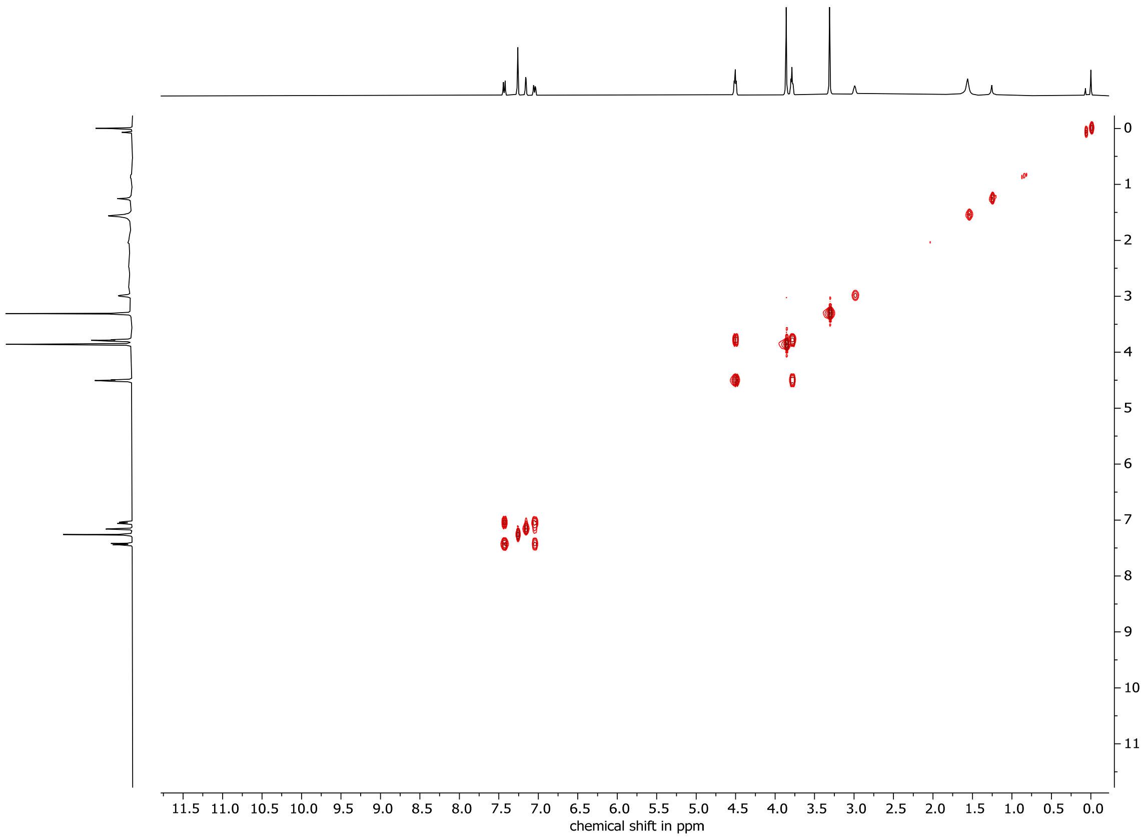

Figure S9. COSY (^1H, ^1H) NMR spectrum of compound **2*m*** in $CDCl_3$.

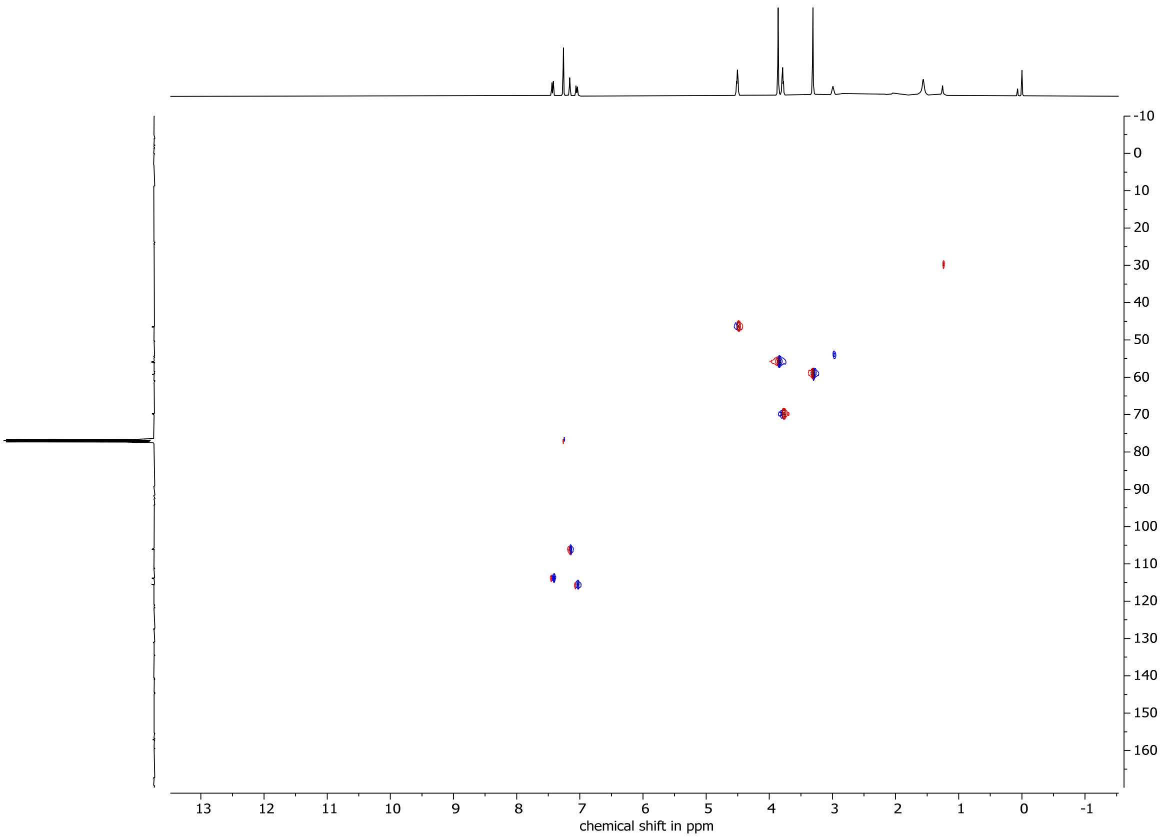

Figure S10. HSQC (^1H, ^{13}C) NMR spectrum of compound **2*m*** in $CDCl_3$.

Compound $\mathbf{2}_{p}$

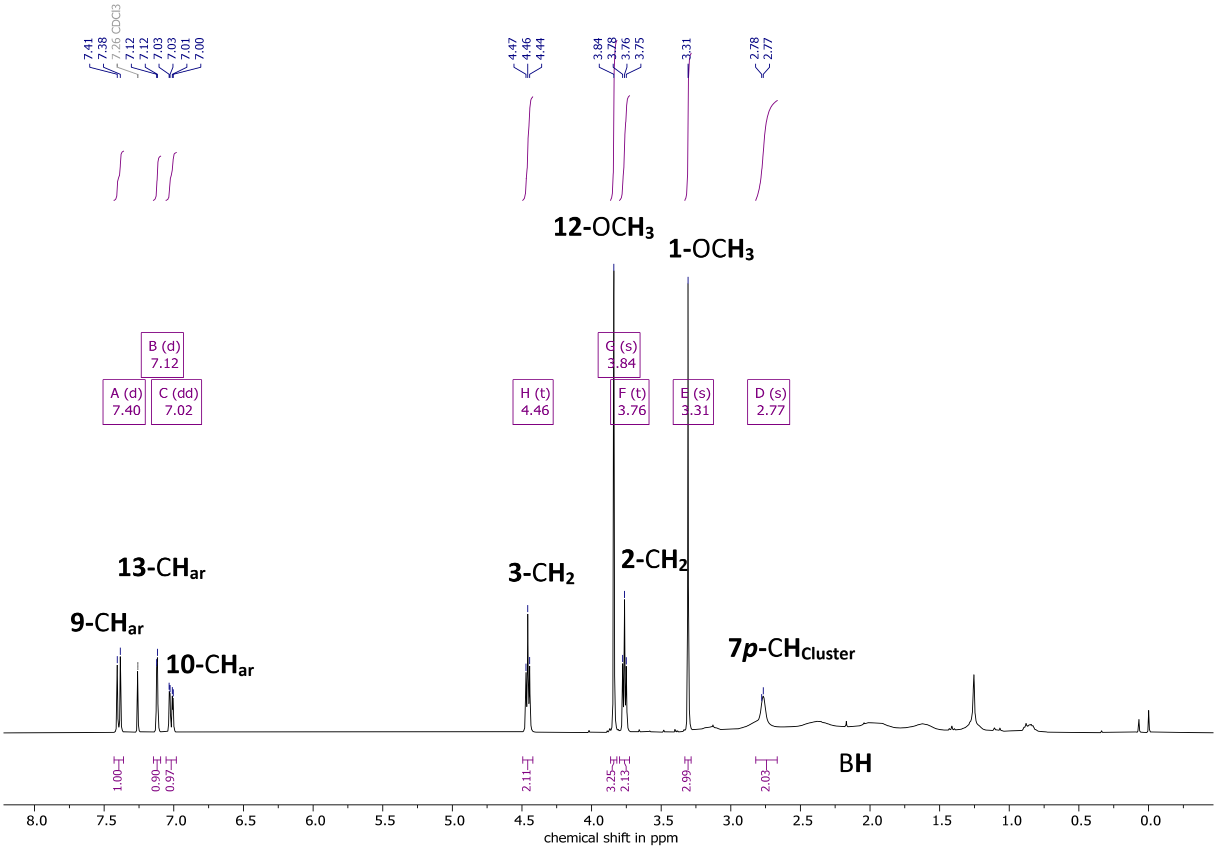

Figure S11. ^{1}H NMR spectrum of compound $\mathbf{2}_{p}$ in $CDCl_3$

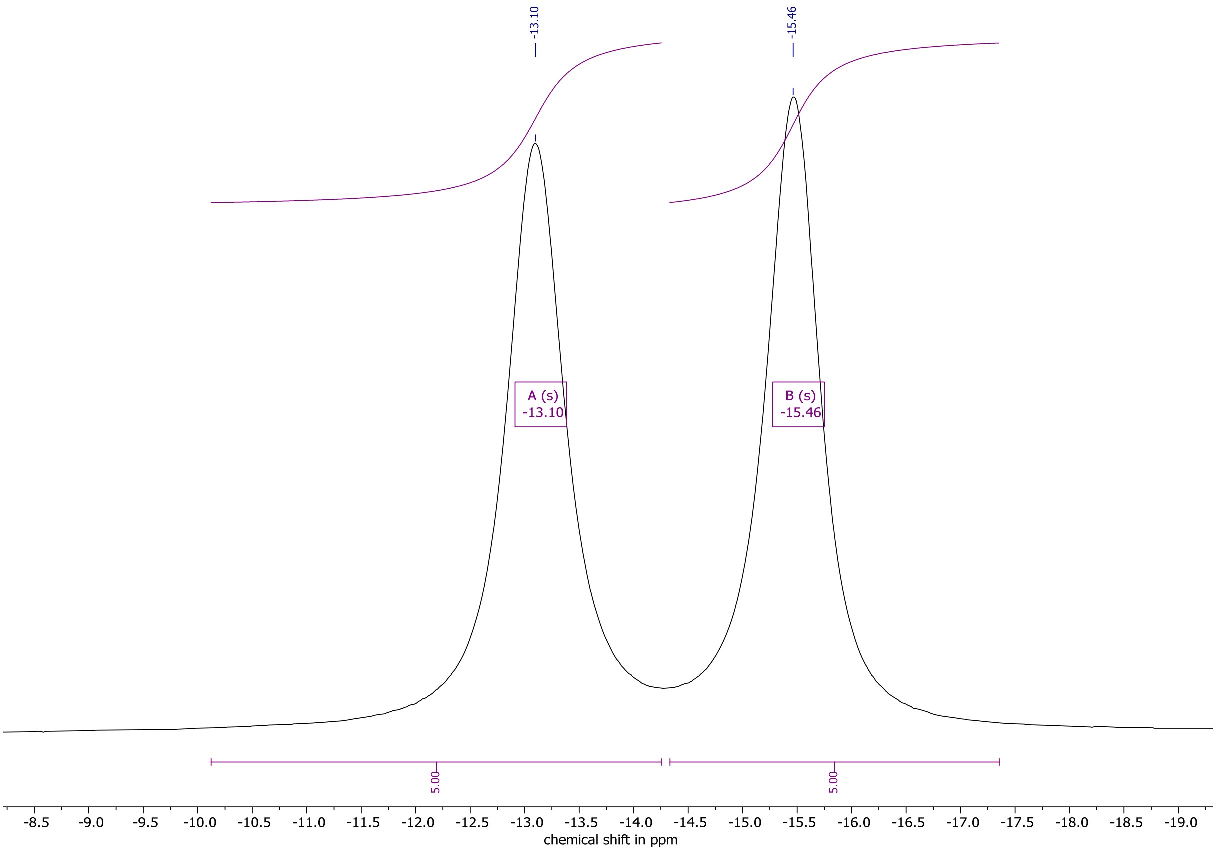

Figure S12. ^{11}B{^{1}H} NMR spectrum of compound $\mathbf{2}_{p}$ in $CDCl_3$.

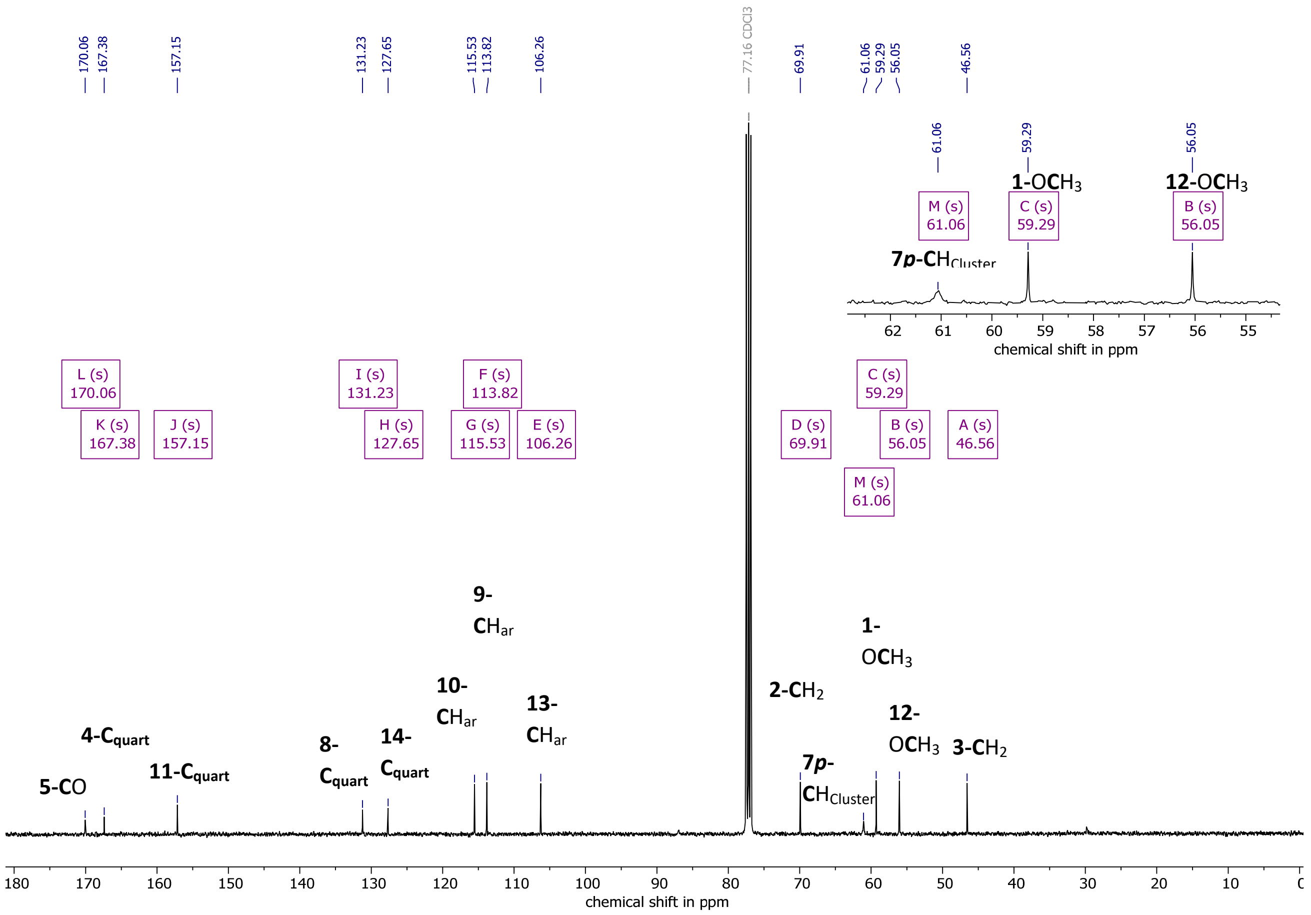

Figure S13. $^{13}C\{^{1}H\}$ NMR spectrum of compound **2*p*** in $CDCl_3$.

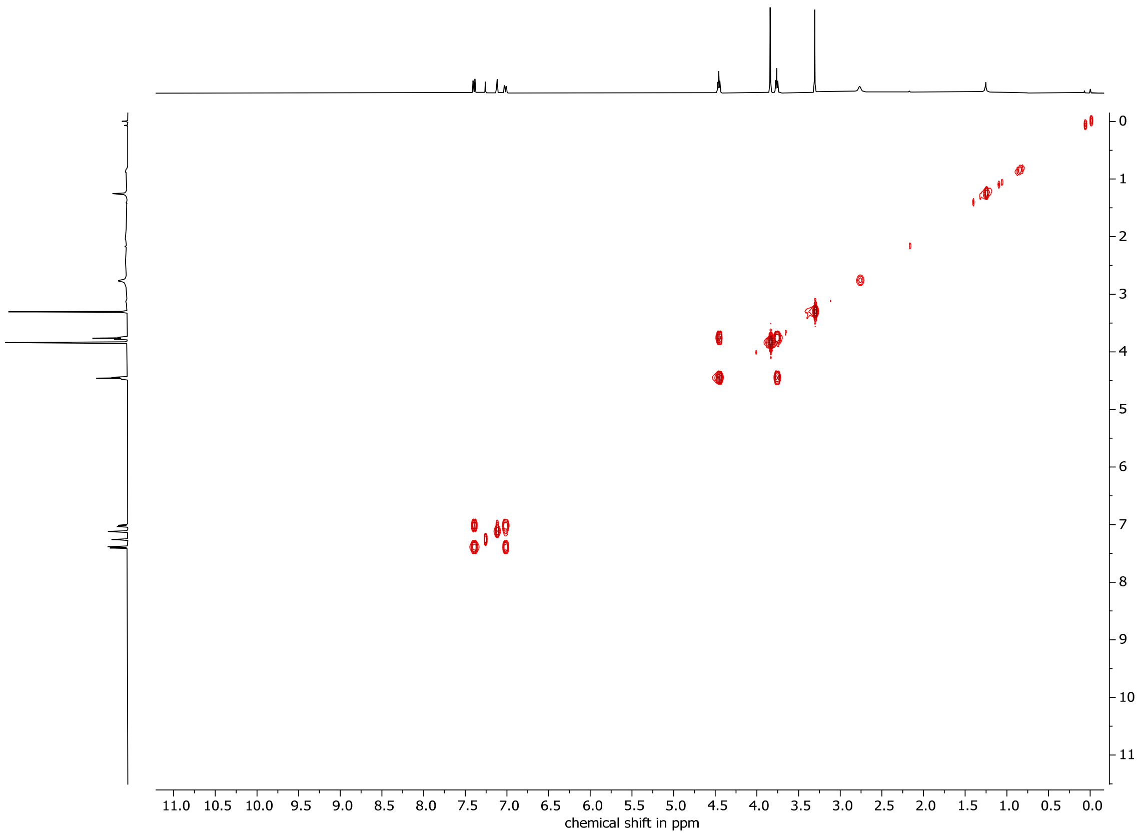

Figure S14. COSY (^{1}H, ^{1}H) NMR spectrum of compound **2*p*** in $CDCl_3$.

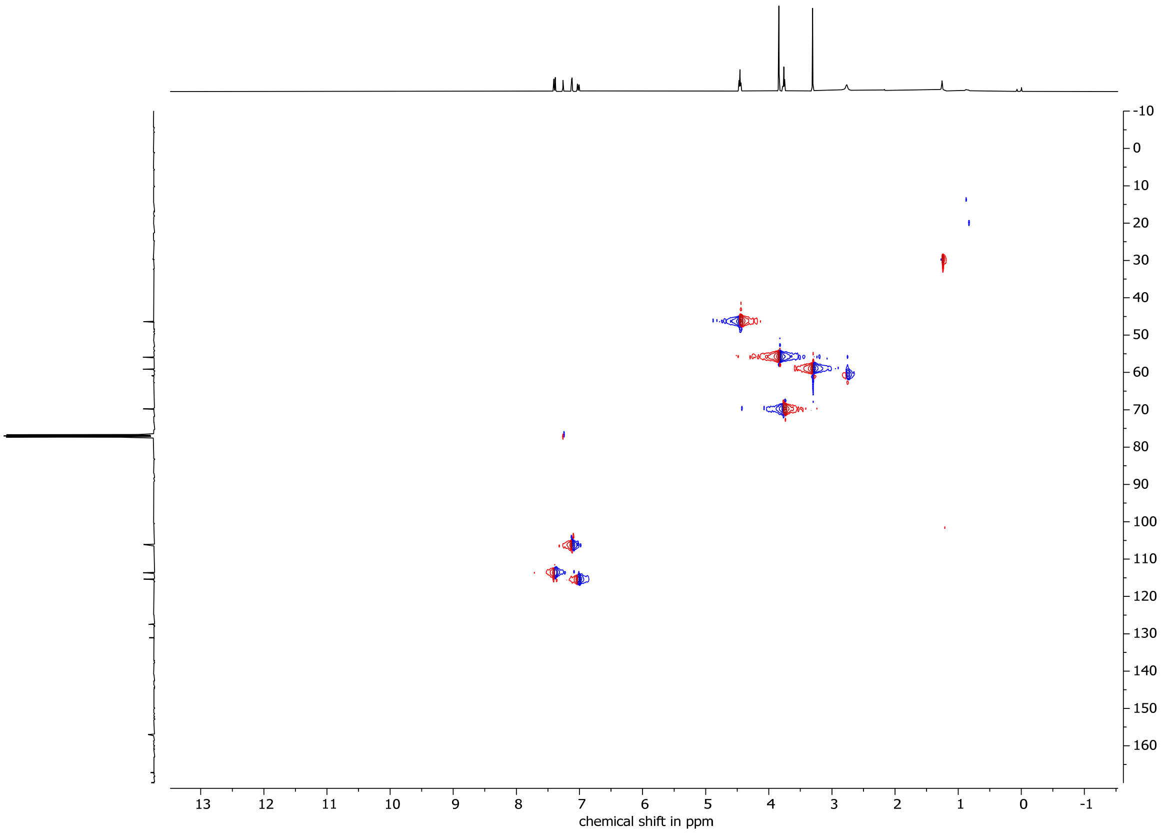

Figure S15. HSQC (^{1}H, ^{13}C) NMR spectrum of compound **2*p*** in $CDCl_3$.

3 HR-ESI Mass Spectra of Compounds 2_o, 2_m and 2_p

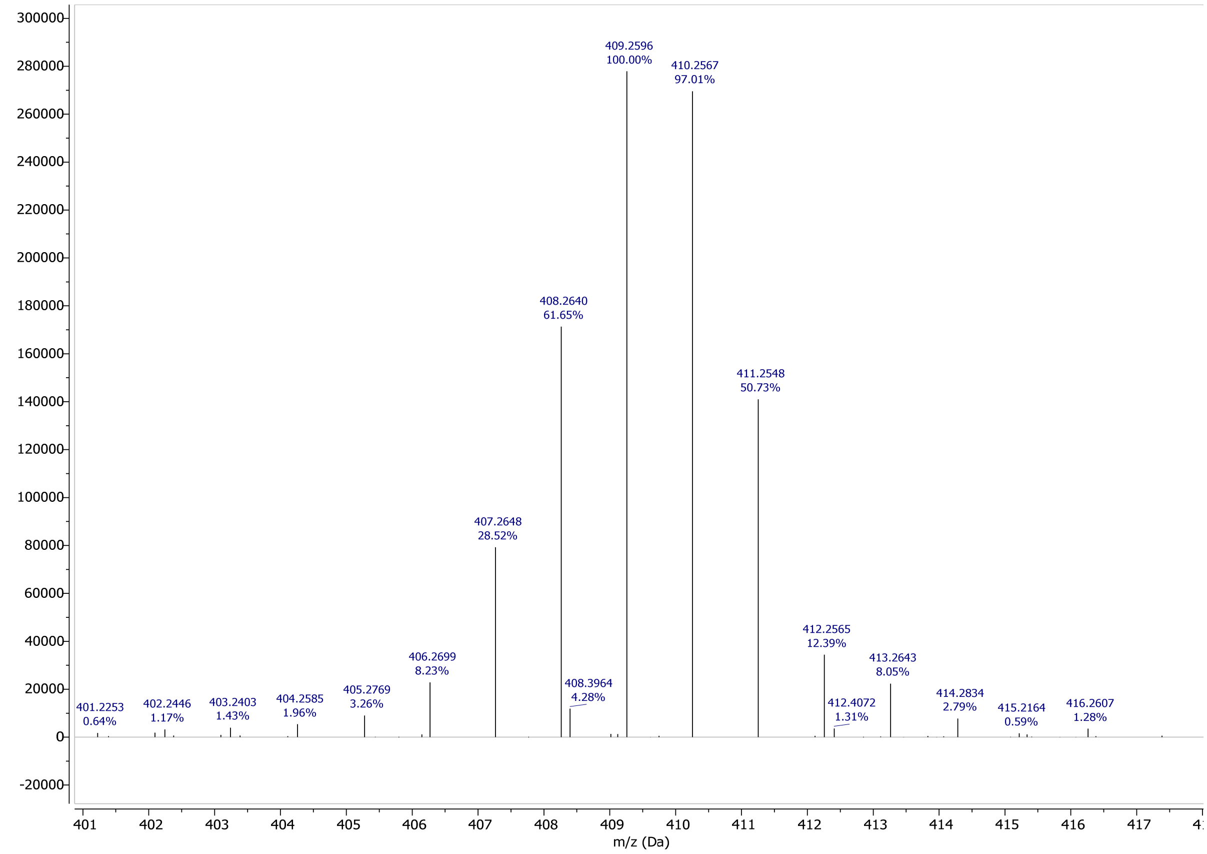

Figure S16. Section of the HRMS (ESI+) of compound $\mathbf{2}_{\boldsymbol{o}}$ in CH_3CN.

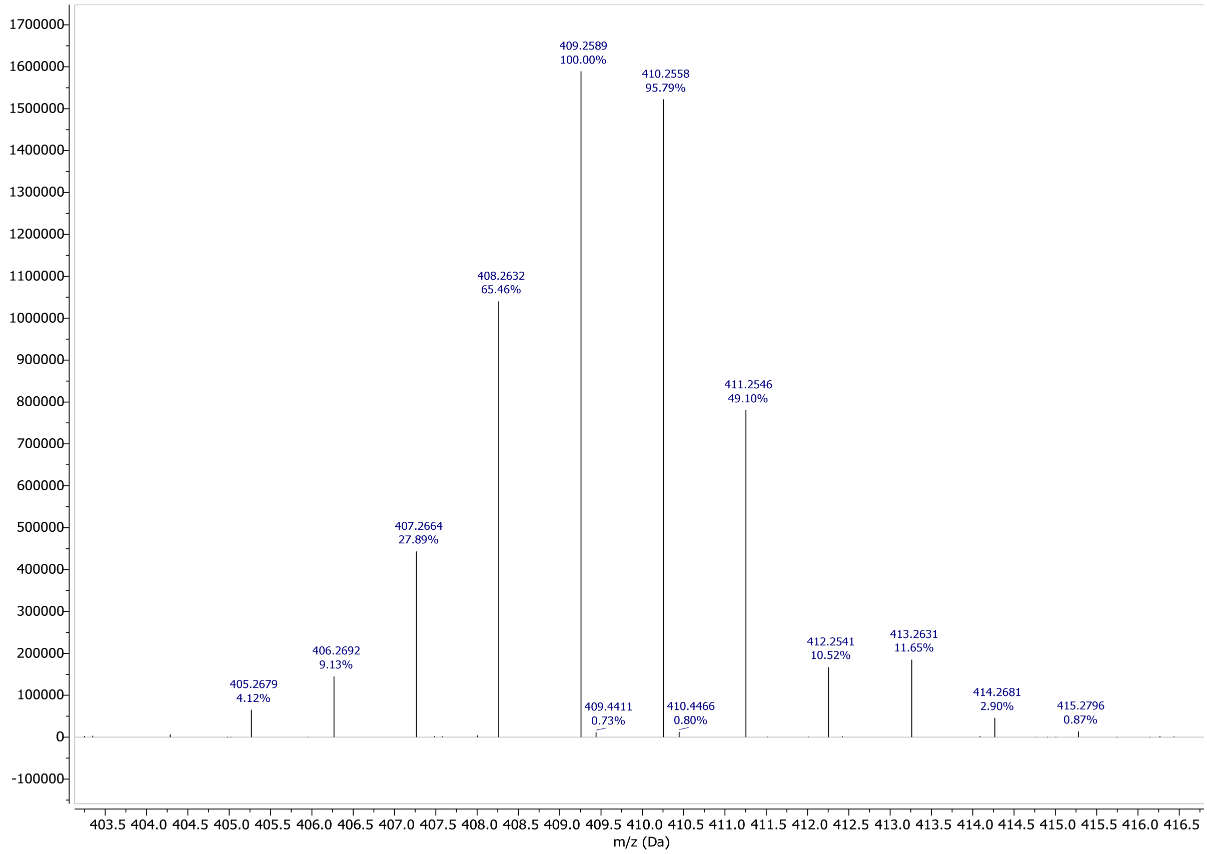

Figure S17. Section of the HRMS (ESI+) of compound $\mathbf{2}_{\boldsymbol{m}}$ in CH_3CN.

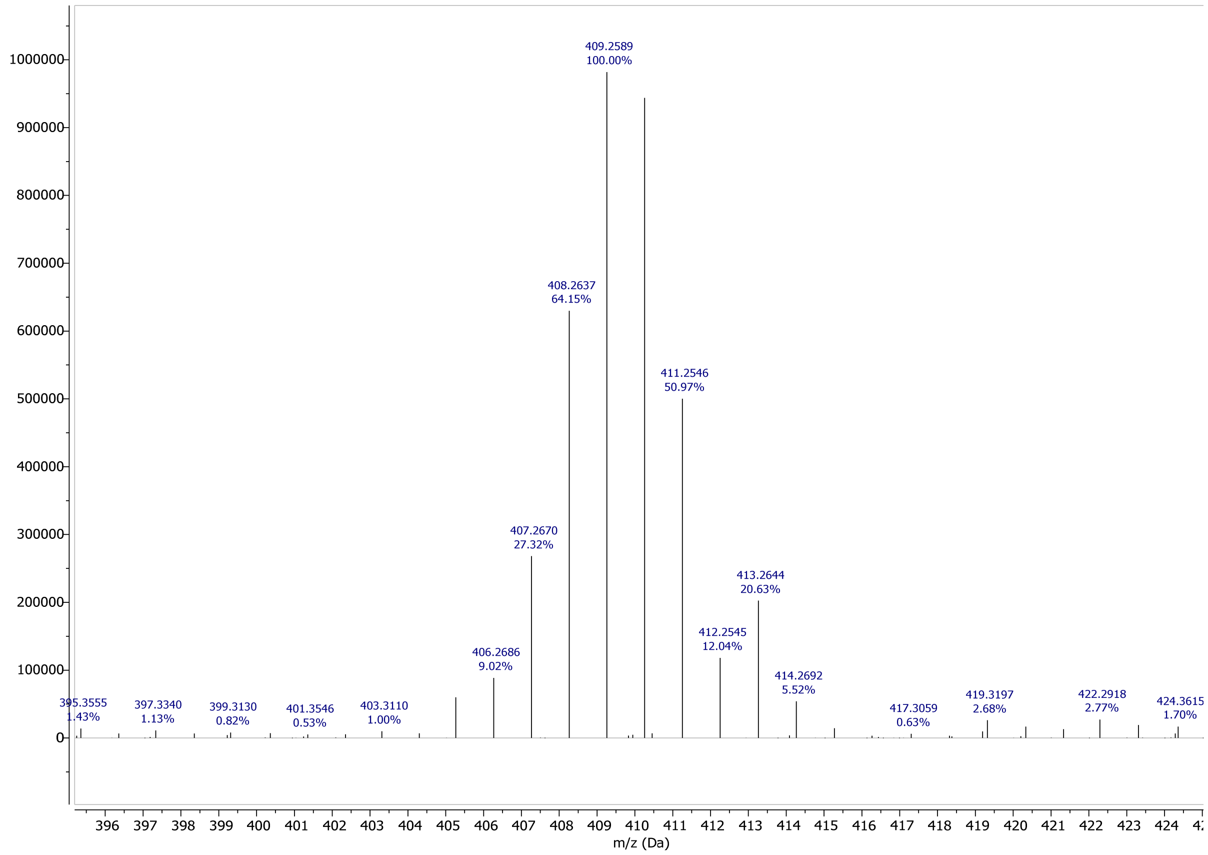

Figure S18. Section of the HRMS (ESI+) of compound $\mathbf{2_p}$ in CH_3CN.

4 Determination of HPLC Purity of Compounds 2$_o$, 2$_m$ and 2$_p$

The purity was determined with HPLC-MS on a RP column. All compounds were dissolved in CH_3CN and prior to the sample, a blank sample was measured. All compounds had a purity >95%. The double-signal appeared because of the column used. A compound relation was excluded, since the signals could not be resolved using a variation of the gradient.

Compound **2$_o$**

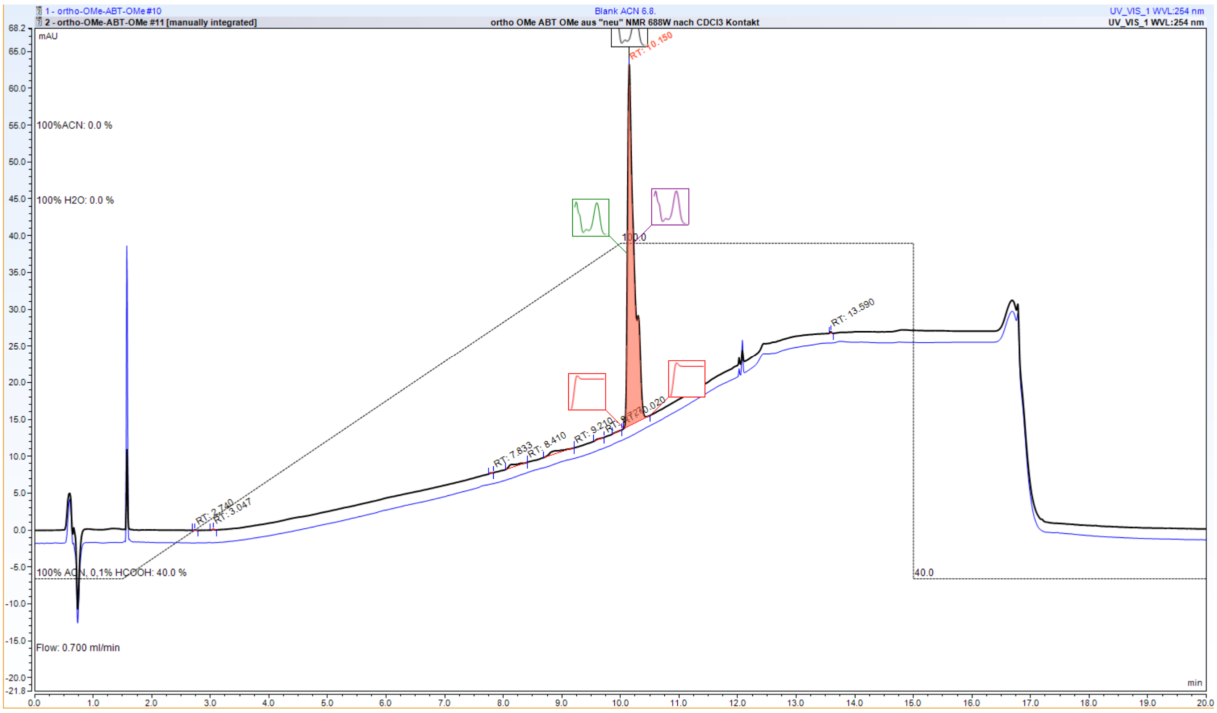

Figure S19. RP-HPLC chromatogram of blank (CH_3CN) and compound **2$_o$**, retention time: 10.2 min.

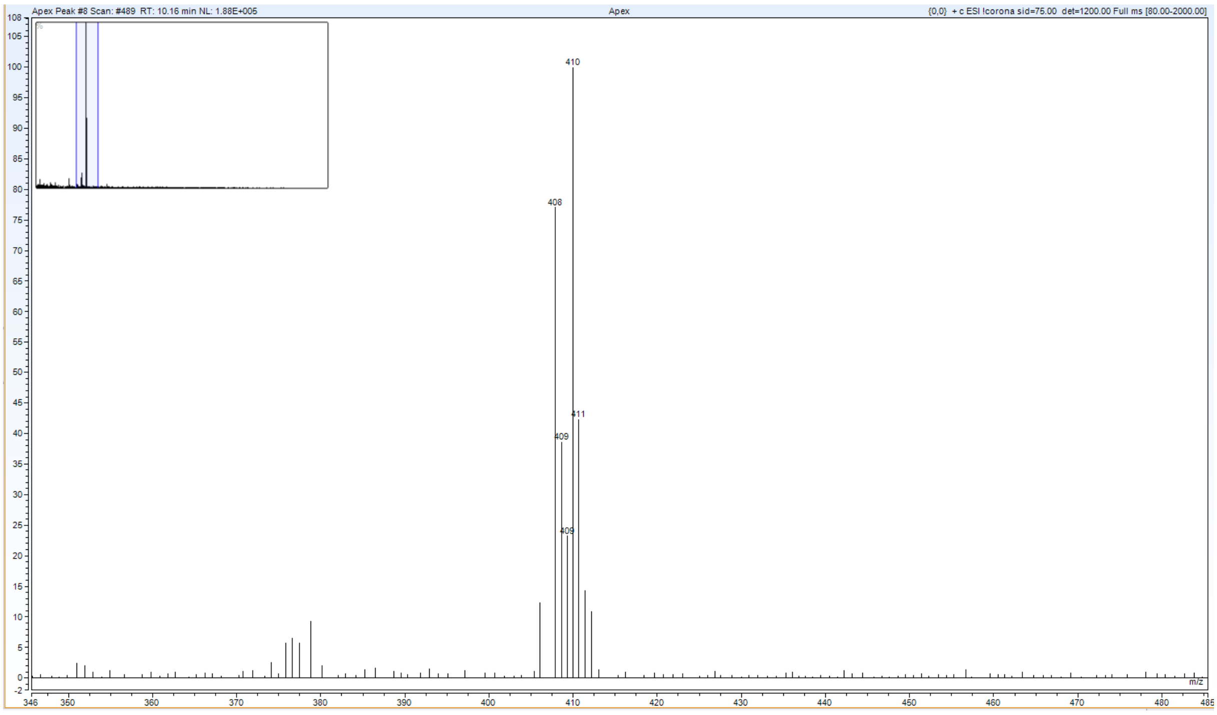

Figure S20. MS(+) of compound **2$_o$**, retention time: 10.2 min.

Compound 2_m

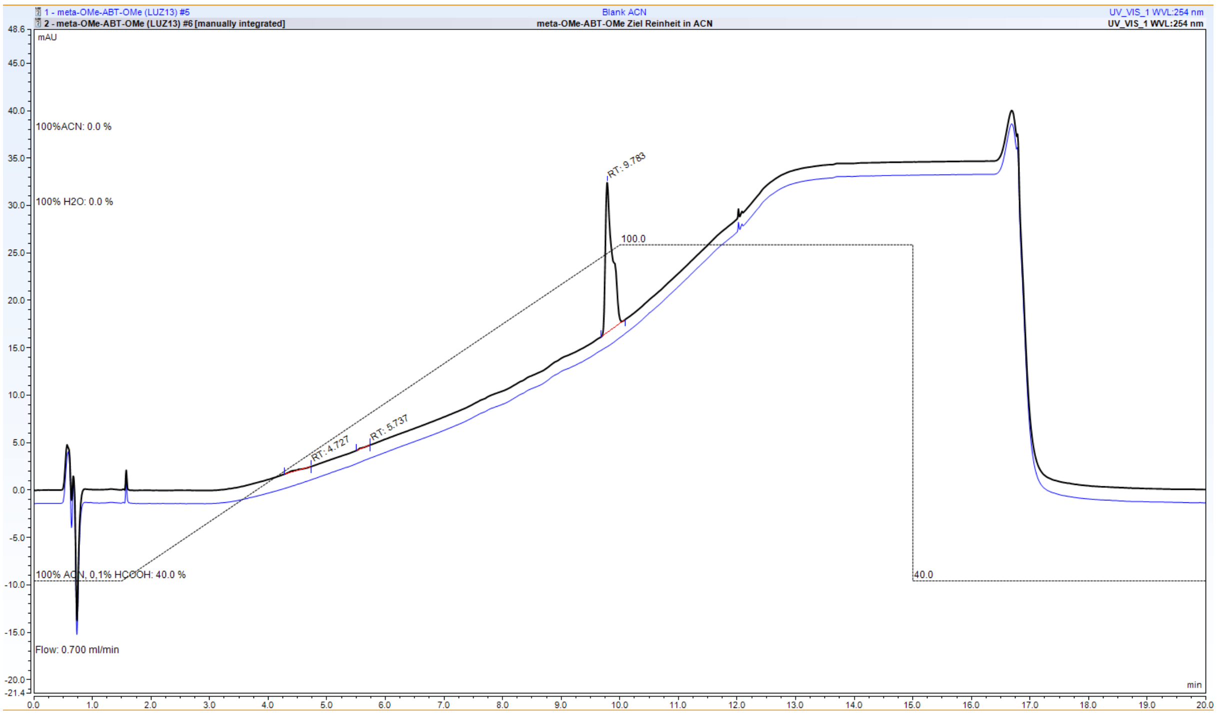

Figure S21. RP-HPLC chromatogram of blank (CH_3CN) and compound **2*m***, retention time: 9.8 min.

Compound 2_p

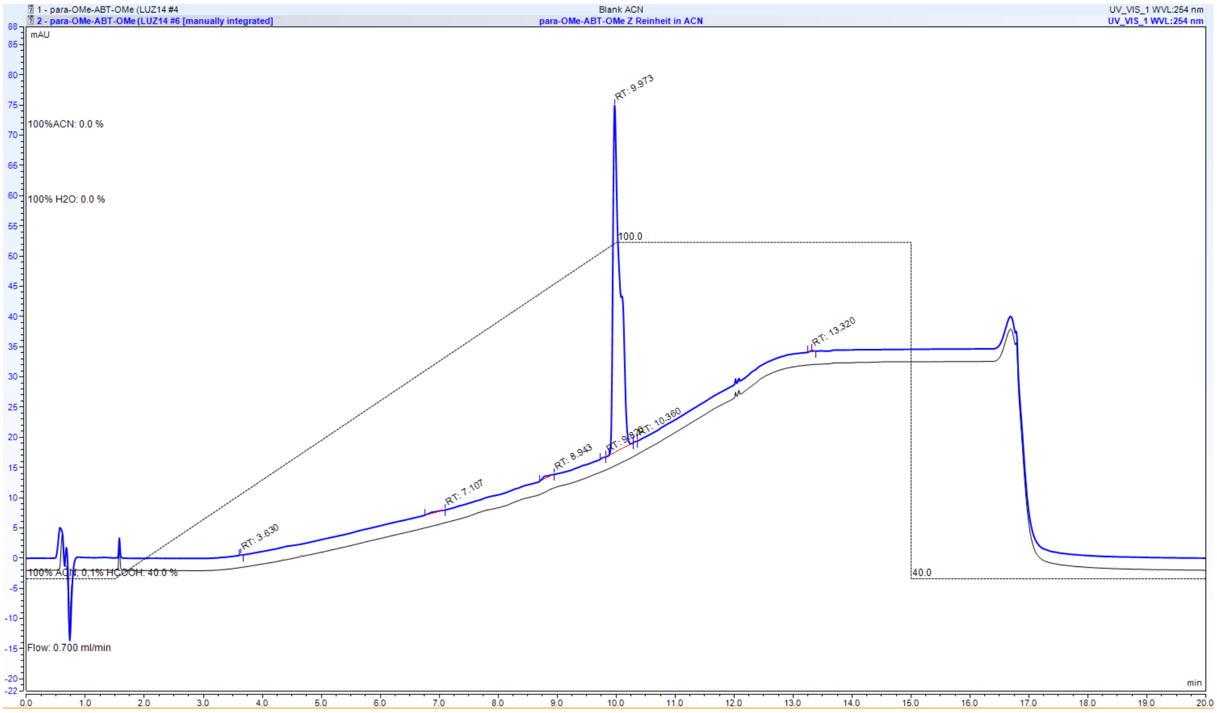

Figure S22. RP-HPLC chromatogram of blank (CH_3CN) and compound **2*p***, retention time: 10.0 min.

5 Determination of the Stability of Compounds 2_o, 2_m and 2_p by HPLC

The stability was determined with HPLC-MS on an RP column. The samples have been measured in DMSO/H_2O (1:1, *v*/*v*). The measurements have been started directly after addition of H_2O to the respective compounds dissolved in DMSO. Prior to each target compound, a blank sample (pure DMSO/H_2O, 1:1, *v*/*v*) was measured. The double-signals are observed due to the column used. A variation of the gradient could not resolve the double-signals.

Compound **2*o***

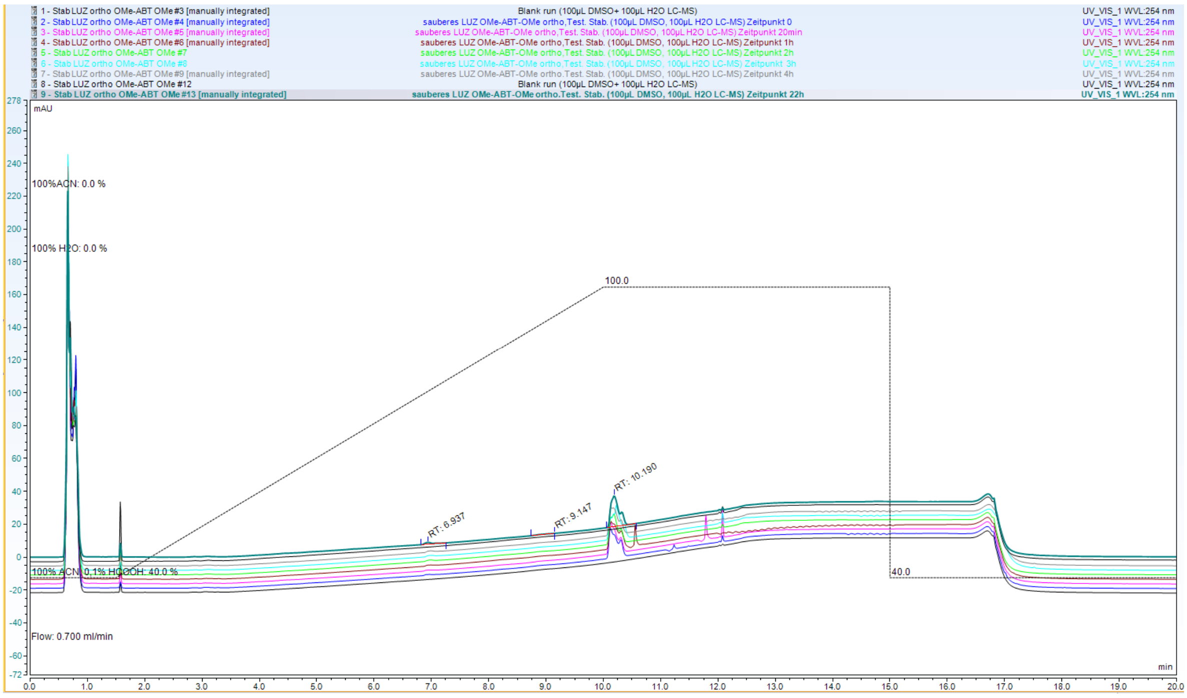

Figure S23. RP-HPLC chromatograms of blank (DMSO/H_2O, black) and compound **2*o***, retention time: 10.2 min, purity after 22 h:~ 95%

Compound **2*m***

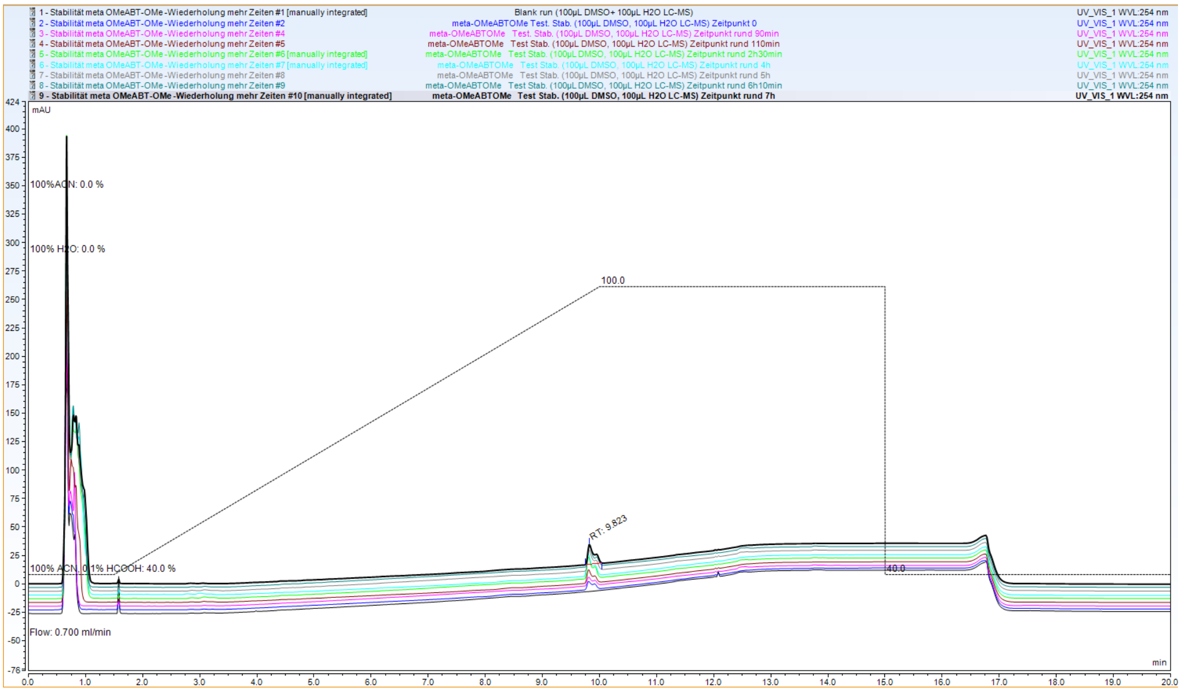

Figure S24. RP-HPLC chromatograms of blank (DMSO/H_2O, black) and compound **2*m***, retention time: 9.8 min, purity after 7 h: unchanged.

Compound **2_p**

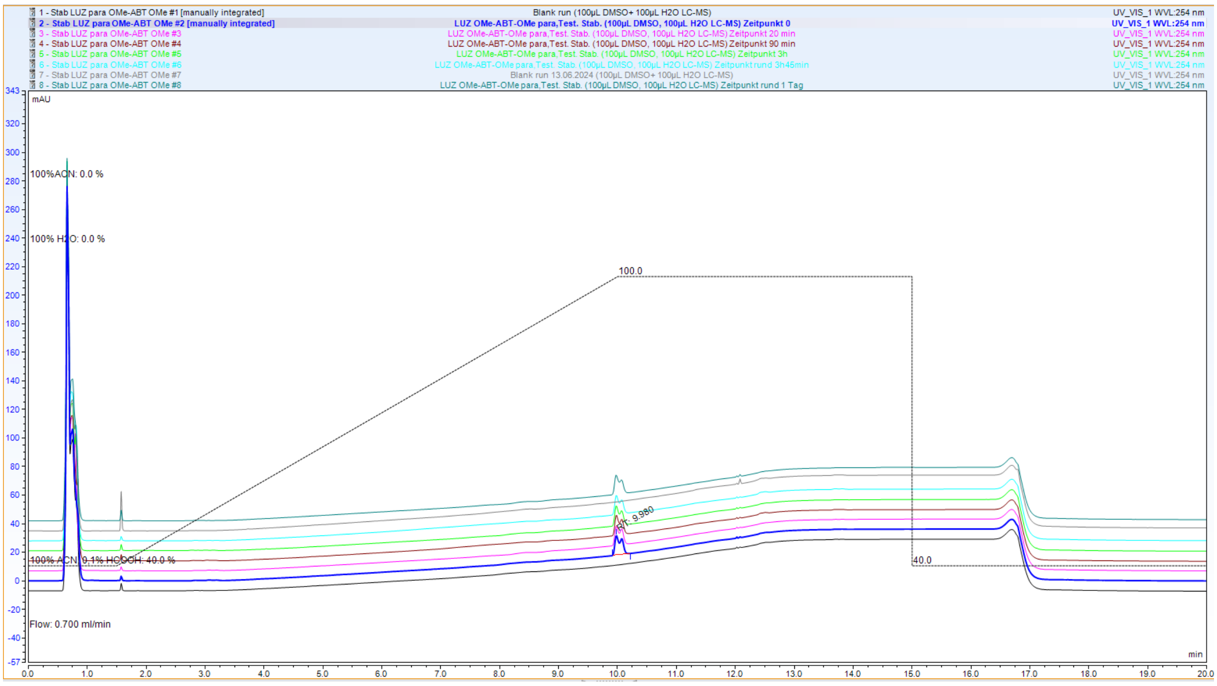

Figure S25. RP-HPLC chromatograms of blank (DMSO/H_2O, black, grey) and compound **2_p**, retention time: 10.0 min, purity after 1 d: unchanged.

6 Docking Studies of Compounds 2_o, 2_m and 2_p

The structures of **2_o, 2_m and 2_p** were constructed *in silico* and optimised using the semiempirical PBEh-3c method developed by the ORCA team.[5] Molecular docking was performed using AutoDockTools4[6] with the Lamarckian Genetic Algorithm.[7] The force-field parameters for boron atoms were manually added to the AutoDockTools4 parameter file.

The protein structure (PDB ID: 5ZTY)[8] was obtained from the Protein Data Bank (PDB) and originally contained the ligand *N*-(adamantan-1-yl)-1-(5-hydroxypentyl)-4-methyl-5-phenyl-1*H*-pyrazole-3-carboxamide. The docking site was selected based on the position of this ligand within the binding pocket. Prior to docking, the ligand and water molecules were removed, and the protein structure was protonated using Reduce software.[9]

For docking, water molecules were eliminated, and non-polar hydrogen atoms were merged. The docking grid box was set with the parameters below:

52 x 46 x 50 centre at (8.12, 3.367, -60.146),

aligning with the ligand-binding domain (LBD).

The following docking parameters were used:

- Number of hybrid GA-LS runs: 100
- Population size: 150
- Maximum number of energy evaluations: 25,000,000
- Top individuals surviving to the next generation: 1
- Gene mutation rate: 0.02
- Crossover rate: 0.8
- Mean of Cauchy distribution for gene mutation: 0.0
- Variance of Cauchy distribution for gene mutation: 1.0

7 Chemical Structures of Compounds SR141716A and WIN55212-2

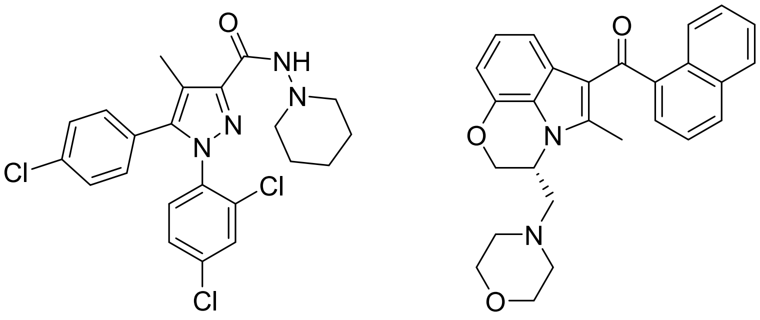

SR141716A **WIN55212-2**

Figure S26. Chemical structures of compounds **SR14176A** (CB_1R antagonist/inverse agonist) and **WIN55212-2** (CB_2R agonist).

References

(1) Choi, S.; Byun, Y. Synthesis of sterically-hindered 1,7-dicarba-*closo*-dodecarborane thiourea analogs. *J. Organomet.Chem.* **2013**, *733*, 49–52. DOI: 10.1016/j.jorganchem.2013.03.003.

(2) Scholz, M.; Blobaum, A. L.; Marnett, L. J.; Hey-Hawkins, E. *Ortho*-carbaborane derivatives of indomethacin as cyclooxygenase (COX)-2 selective inhibitors. *Bioorg. Med. Chem.* **2012**, *20* (15), 4830–4837. DOI: 10.1016/j.bmc.2012.05.063.

(3) Kasar, R. A.; Knudsen, G. M.; Kahl, S. B. Synthesis of 3-Amino-1-carboxy-*o*-carborane and an Improved, General Method for the Synthesis of All Three C-Amino-C-carboxycarboranes. *Inorg. Chem.* **1999**, *38* (12), 2936–2940. DOI: 10.1021/ic990037o.

(4) Aly, M. W.; Ludwig, F.-A.; Deuther-Conrad, W.; Brust, P.; Abadi, A. H.; Moldovan, R.-P.; Osman, N. A. Development of fluorinated and methoxylated benzothiazole derivatives as highly potent and selective cannabinoid CB2 receptor ligands. *Bioorg. Chem.* **2021**, *114*, 105191. DOI: 10.1016/j.bioorg.2021.105191.

(5) Neese, F. Software update: the ORCA program system, version 4.0. *WIREs Comput. Mol. Sci.* **2018**, *8* (1). DOI: 10.1002/wcms.1327.

(6) Morris, G. M.; Huey, R.; Lindstrom, W.; Sanner, M. F.; Belew, R. K.; Goodsell, D. S.; Olson, A. J. AutoDock4 and AutoDockTools4: Automated docking with selective receptor flexibility. *J. Comput. Chem.* **2009**, *30* (16), 2785–2791. DOI: 10.1002/jcc.21256.

(7) Morris, G. M.; Goodsell, D. S.; Halliday, R. S.; Huey, R.; Hart, W. E.; Belew, R. K.; Olson, A. J. Automated docking using a Lamarckian genetic algorithm and an empirical binding free energy function. *J. Comput. Chem.* **1998**, *19* (14), 1639–1662. DOI: 10.1002/(SICI)1096-987X(19981115)19:14<1639:AID-JCC10>3.0.CO;2-B.

(8) Li, X.; Hua, T.; Vemuri, K.; Ho, J.-H.; Wu, Y.; Wu, L.; Popov, P.; Benchama, O.; Zvonok, N.; Locke, K.; Qu, L.; Han, G. W.; Iyer, M. R.; Cinar, R.; Coffey, N. J.; Wang, J.; Wu, M.; Katritch, V.; Zhao, S.; Kunos, G.; Bohn, L. M.; Makriyannis, A.; Stevens, R. C.; Liu, Z.-J. Crystal Structure of the Human Cannabinoid Receptor CB2. *Cell* **2019**, *176* (3), 459-467.e13. DOI: 10.1016/j.cell.2018.12.011.

(9) Word, J.; Lovell, S. C.; Richardson, J. S.; Richardson, D. C. Asparagine and glutamine: using hydrogen atom contacts in the choice of side-chain amide orientation. *J. Mol. Biol.* **1999**, *285* (4), 1735–1747. DOI: 10.1006/jmbi.1998.2401.
